# Proteases Shape the *Chlamydomonas* Secretome: Comparison to Classical Neuropeptide Processing Machinery

**DOI:** 10.3390/proteomes6040036

**Published:** 2018-09-23

**Authors:** Raj Luxmi, Crysten Blaby-Haas, Dhivya Kumar, Navin Rauniyar, Stephen M. King, Richard E. Mains, Betty A. Eipper

**Affiliations:** 1 Department of Neuroscience, University of Connecticut Health Center, Farmington, CT 06030-3401, USA; luxmi@uchc.edu (R.L.); mains@uchc.edu (R.E.M.); 2 Department of Biology, Brookhaven National Laboratory, Upton, NY 11973-5000, USA; cblaby@bnl.gov; 3 Department of Molecular Biology and Biophysics, University of Connecticut Health Center, Farmington, CT 06030-3305, USA; dhivya.kumar@ucsf.edu (D.K.); king@uchc.edu (S.M.K.); 4 W.M. Keck Biotechnology Resource Laboratory, Yale University, New Haven, CT 06511-6624, USA; navin.rauniyar@yale.edu

**Keywords:** peptidylglycine α-amidating monooxygenase, cilia, mating, signal peptide, prohormone convertase, carboxypeptidase, matrix metalloproteinase, subtilisin, pherophorin

## Abstract

The recent identification of catalytically active peptidylglycine α-amidating monooxygenase (PAM) in *Chlamydomonas reinhardtii*, a unicellular green alga, suggested the presence of a PAM-like gene and peptidergic signaling in the last eukaryotic common ancestor (LECA). We identified prototypical neuropeptide precursors and essential peptide processing enzymes (subtilisin-like prohormone convertases and carboxypeptidase B-like enzymes) in the *C. reinhardtii* genome. Reasoning that sexual reproduction by *C. reinhardtii* requires extensive communication between cells, we used mass spectrometry to identify proteins recovered from the soluble secretome of mating gametes, and searched for evidence that the putative peptidergic processing enzymes were functional. After fractionation by SDS-PAGE, signal peptide-containing proteins that remained intact, and those that had been subjected to cleavage, were identified. The *C. reinhardtii* mating secretome contained multiple matrix metalloproteinases, cysteine endopeptidases, and serine carboxypeptidases, along with one subtilisin-like proteinase. Published transcriptomic studies support a role for these proteases in sexual reproduction. Multiple extracellular matrix proteins (ECM) were identified in the secretome. Several pherophorins, ECM glycoproteins homologous to the *Volvox* sex-inducing pheromone, were present; most contained typical peptide processing sites, and many had been cleaved, generating stable N- or C-terminal fragments. Our data suggest that subtilisin endoproteases and matrix metalloproteinases similar to those important in vertebrate peptidergic and growth factor signaling play an important role in stage transitions during the life cycle of *C. reinhardtii*.

## 1. Introduction

**Figure 1:**
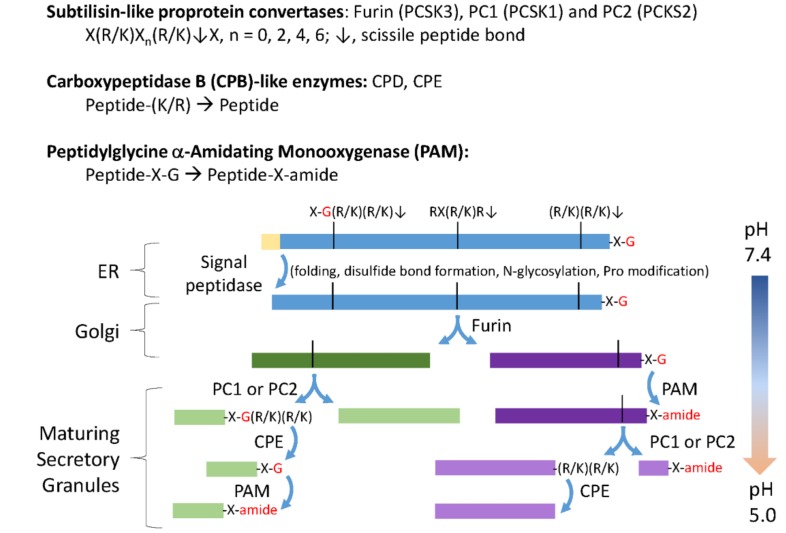
Classical neuropeptide precursors and processing enzymes. The biosynthesis and post-translational processing of neuropeptide precursors from organisms as diverse as human and *Hydra* employs a common set of subcellular organelles and processing enzymes. The reactions catalyzed by subtilisin-like prohormone convertases, carboxypeptidase B (CPB)-like enzymes and peptidylglycine α-amidating monooxygenase (PAM) are shown. Endoplasmic reticulum (ER) entry requires an N-terminal signal peptide, which is quickly removed. As for other secreted proteins, N-glycosylation, disulfide bond formation, proline hydroxylation, and proline isomerization are accomplished before transit through the Golgi complex. A family of subtilisin-like endoproteases, referred to as prohormone convertases (PCs), catalyze a series of ordered endoproteolytic cleavages, with furin (PCSK3), PC1 (PCSK1), and PC2 (PCSK2) playing especially important roles in many neurons and endocrine cells. Endoproteolytic cleavage is controlled, in large part, by the pH of the luminal compartment, with furin active in the *trans*-Golgi network and endocytic compartments, and PC1 and PC2 more active in the low pH environment encountered in immature and mature secretory granules. CPB-like enzymes (CPE and CPD) remove the C-terminal Lys and Arg residues produced by furin, PC1, and PC2. The amidating enzyme, PAM, requires only a C-terminal Gly residue to amidate the penultimate residue (–X–amide); in the presence of adequate copper, ascorbate, and molecular oxygen, PAM can function throughout the biosynthetic pathway [6].

Identification of the enkephalins as endogenous ligands for opioid receptors led to the successful description of hundreds of additional bioactive peptides in the nervous systems of species as diverse as *Drosophila*, *Caenorhabditis elegans*, and *Hydra* [1,2,3]. Like proinsulin and proopiomelanocortin, the precursors to these neuropeptides have N-terminal signal sequences, multiple potential paired basic amino acid endoproteolytic cleavage sites, potential amidation sites, generally lack recognized domains, and often contain multiple copies of similar peptides (Figure 1). Bioinformatic criteria for the identification of potential neuropeptide precursors have been successfully applied to many systems [4,5].

With the availability of genomic and transcriptomic data from a diverse array of organisms, it quickly became clear that “neuropeptide precursors” were quite prevalent in species lacking recognizable neurons or endocrine cells. *Trichoplax*, a basal, multicellular animal, lacks muscles and neurons [7,8,9]. Despite this, its genome encodes many of the proteins that define the nervous system: candidate voltage-gated ion channels, SNARE proteins, and neuropeptide precursors are present. *Trichoplax* use the beating of ventrally located cilia to glide over surfaces, pausing to secrete the enzymes needed to feed on algae. Ciliated gland cells produce peptide-containing secretory granules that may be used to control locomotion and digestion. Single celled eukaryotes exhibit behaviors such as phototaxis and chemotaxis, leading to the suggestion that the first steps of nervous system evolution occurred in a ciliated organism, with neural circuits evolving to control locomotor cilia [10]. For example, amidated peptides synthesized in sensory neurons regulate the swimming of *Platynereis dumerilii* larvae by controlling ciliary beat frequency [11].

The enzymes involved in converting neuropeptide precursors into bioactive peptide products are highly conserved (Figure 1). All must function within the secretory pathway, where luminal pH plays a key role in controlling precursor cleavage and product storage in secretory granules. Like other secreted proteins, neuropeptide precursors often contain essential disulfide bonds, are modified by N- and O-linked glycosylation, and are phosphorylated. A set of calcium-dependent subtilisin-like endoproteases, CPB-like enzymes, and PAM, are generally regarded as reliable markers for neuropeptide-producing cells [12,13,14,15]. The discovery of a fully functional PAM protein in *Chlamydomonas reinhardtii* suggested the presence of “neuropeptides” in *C. reinhardtii*, and in the last eukaryotic common ancestor. Secretory granules have not been observed in *C. reinhardtii* and CrPAM is localized to the Golgi and to the ciliary membrane [16]. Cells in which expression of CrPAM was reduced were unable to assemble cilia that extended beyond the transition zone [17]. Using a bioinformatics approach, we searched for and found putative preproneuropeptides, and a complete set of peptide-processing enzymes encoded by the *C. reinhardtii* genome.

Sexual reproduction, which began in the last eukaryotic common ancestor [18], is triggered in *C. reinhardtii* by nutrient restriction. Based on studies in multiple systems, this process is likely to involve peptidergic signaling [2,4] and regulated secretion [19]. Many of the interactions and signaling pathways involved in *C. reinhardtii* mating have been well characterized, and detailed transcriptomic data for specific stages are available [20,21]. Therefore, to evaluate the functional role(s) of “neuropeptide” processing machinery in a unicellular eukaryote, we undertook an analysis of the mating secretome of *C. reinhardtii*. Using differential centrifugation, we separated mating ectosomes, small vesicles derived from the cilia, from the soluble mating secretome. We focus here on our analysis of the proteases and cleaved products identified in the soluble mating secretome. 

## 2. Material and Methods

### 2.1. Strains and Growth Conditions

CC124 (mating type *minus*) and CC125 (mating type *plus*) *C. reinhardtii* strains (Chlamydomonas Resource Center) were cultured in R-medium aerated with 95% air and 5% CO_2_ under a 12 h light/12 h dark cycle at 22 °C. Vegetative cells were grown for 5 days under these culture conditions. To induce gametogenesis, vegetative cells were harvested, washed, and resuspended in nitrogen-deficient minimal medium (M-N/5 medium) for 16–20 h [22]. The mating competency of the gametes was assessed by mixing mating type *minus* and *plus* gametes and microscopically verifying >80% agglutination. 

### 2.2. Preparation of Soluble Mating Secretome

In preliminary experiments, CC124 and CC125 gametes checked for high mating efficiency were mixed together for 1 h with gentle aeration at 22 °C. Following removal of cells (1600× *g* for 5 min) and debris (20,000× *g* for 10 min), the mating medium was filtered through a 0.22 µm filter to remove particulate material. The filtrate was concentrated 7-fold using a tangential flow filter system with a 2 kDa cutoff (Sartorius Vivaflow 200, Hydrosart, 2K; Thermo Fisher Scientific, Waltham, MA, USA). The concentrated mating medium was subjected to SDS-PAGE; after visualization by silver staining (Pierce, SilverSnap kit; Thermo Fisher Scientific, Rockford, IL, USA), the gel lane was excised. Gel fragments containing high and low molecular weight proteins were separately subjected to in-gel trypsin digestion, essentially as described below. Tandem mass spectrometry was carried out at the Proteomics and Mass Spectrometry Facility at the University of Massachusetts Medical School. The sample analyzed in this preliminary experiment contained both ectosomes and soluble proteins; in subsequent analyses, additional centrifugation steps yielded ectosomes and the soluble secretome, which were analyzed separately.

Gametes of both mating types (*plus* and *minus*) were washed and resuspended in 10 mL of fresh nitrogen-free medium at a density of 8–10 × 10^6^ cells/mL. An equal number of mating type *plus* and *minus* gametes were mixed; after a 1 h incubation, the cultures were centrifuged at 1600× *g* as described above. These supernatants were then centrifuged at 20,000× *g* for 30 min at 4 °C to pellet cell debris. The resulting supernatants were next centrifuged at 200,000× *g* for 60 min at 4 °C to sediment all particulate material, including extracellular vesicles. These final supernatants are referred to as the soluble mating secretome. A protease inhibitor cocktail (Roche, cOmplete ULTRA Tablets, Cat. No. 05 892 791 001) and phenylmethylsulfonyl fluoride (final concentration 0.3 mg/mL) were added to each sample, which was dispensed in aliquots that were stored at −80 °C. A total of six samples prepared at two different times were subjected to analysis, ultimately yielding Dataset 1 (Samples A, B and C) and Dataset 2 (Samples D, E and F). SDS-PAGE fractionation of 40 μL of the soluble mating secretome yielded bands that were readily visualized using silver staining (Silver Stain for Mass Spectrometry; Thermo Fisher Scientific, Rockford, IL, USA). The low speed pellet (1600× *g* for 5 min) was resuspended in 0.30 mL 20 mM 2-[tris(hydroxymethyl)-methylamino]-ethanesulfonic acid (TES), 10 mM mannitol, pH 7.4 containing 1% TX-100 and a protease inhibitor cocktail; aliquots were assayed for protein content using the bicinchoninic acid assay (Thermo Fisher Scientific, Rockford, IL, USA).

### 2.3. Fractionation for Mass Spectroscopy

Aliquots (40 µL) of each soluble mating secretome were prepared for SDS-PAGE by mixing with 4× Laemmli sample buffer (Bio-Rad, Hercules, CA, USA) and denaturation at 55 °C for 5 min. Each sample was then fractionated on a Criterion TGX 4–15% gradient gel (Bio-Rad, Hercules, CA, USA). Electrophoresis of the first set of samples was stopped when the dye band had traveled 3 cm, and the gel was stained with QC Colloidal Coomassie (Bio-Rad, Hercules, CA, USA), according to the manufacturer’s protocol. Based on molecular weight standards analyzed at the same time, each lane was cut into 10 slices that covered material migrating from the dye band to the top of the gel. Electrophoresis of the second set of samples was stopped when the dye band had traveled 2.5 cm; after staining with QC Colloidal Coomassie, each lane was cut into 4 slices covering the entire molecular weight range. Gel slices were stored frozen in microfuge tubes before preparation for LC-MS/MS analysis after in-gel digestion with trypsin.

### 2.4. Preparation of Vegetative Secretome

Vegetative cells of both mating types (*plus* and *minus*) were grown for 5 days in R-media (500 mL cultures). Cells were washed and resuspended in 5 mL of fresh R-medium. Cells were incubated for 4 h under continuous light with gentle aeration. The soluble vegetative secretome was prepared as described above for the soluble mating secretome. For comparing the vegetative and mating secretomes, the volume of soluble secretome analyzed by gel electrophoresis was adjusted to represent secretion by 200 (vegetative) or 50 (mating) μg of cell protein. 

### 2.5. Mass Spectrometry

Gel bands were cut into small pieces, washed with 250 µL of 50% acetonitrile for 5 min with rocking, then washed with 50% acetonitrile/50 mM NH_4_HCO_3_ for 30 min on a tilt-table. After a final 30 min wash with 50% acetonitrile/10 mM NH_4_HCO_3_, gel fragments were dried using a speed vacuum. Each sample was suspended in 30 µL of 10 mM NH_4_HCO_3_ containing 0.20 µg of digestion grade trypsin (Promega, V5111) and incubated at 37 °C for 16 h. The digestion supernatant was acidified and placed into a vial for LC-MS/MS analysis (5 µL injected).

Data acquisition was conducted on an Orbitrap Fusion Tribrid mass spectrometer. Reversed phase (RP)-LC-MS/MS was performed using a nanoACQUITY UPLC system (Waters Corporation, Milford, MA, USA) connected to an Orbitrap Fusion Tribrid (Thermo Fisher Scientific, San Jose, CA, USA) mass spectrometer. After injection, samples were loaded into a trapping column (nanoACQUITY UPLC Symmetry C18 Trap column, 180 µm × 20 mm) at a flowrate of 5 µL/min and separated using a C18 column (nanoACQUITY column Peptide BEH C18, 75 µm × 250 mm). The compositions of mobile phases A and B were 0.1% formic acid in water and 0.1% formic acid in acetonitrile, respectively. Peptides were eluted with a gradient extending from 3% to 20% mobile phase B in 85 min, and then to 35% mobile phase B in another 35 min at a flowrate of 300 nL/min and a column temperature of 37 °C. The data were acquired with the mass spectrometer operating in a top speed data-dependent acquisition (DDA) mode. An Orbitrap full MS scan was performed in the range of 300–1500 *m*/*z* at a resolution setting of 120,000 with an automatic gain control (AGC) target value of 4 × 10^5^. Iterative isolation (with a 1.6 Thomson unit isolation window and minimum intensity threshold of 5 × 10^4^) and fragmentation by higher-energy collisional dissociation of ions were carried out after the Orbitrap full MS scan. Ions were injected with a maximum injection time of 110 ms and an AGC target of 1 × 10^5^. 

Raw data were processed using Proteome Discoverer software (version 2.1, Thermo Fisher Scientific, San Jose, CA, USA). MS2 spectra were searched using Mascot (Matrix Science, London, UK), which was set up to search against the *Chlamydomonas reinhardtii* database (Creinhardtii_281_v5.5). The search criteria included 10 ppm precursor mass tolerance, 0.02 Da fragment mass tolerance, trypsin enzyme, and maximum missed cleavage sites of two. Dynamic modifications included propionamide on cysteine, oxidation on methionine, deamidation on asparagine and glutamine, and Gly-loss+Amide on C-terminal glycine. Peptide spectral match (PSM) error rates were determined using the target-decoy strategy coupled to Percolator modeling of true and false matches [23,24]. The mass spectrometry proteomics data have been deposited to the ProteomeXchange Consortium via the PRIDE partner repository with the dataset identifier PXD010945.

Scaffold (version Scaffold_4.8.4, Proteome Software Inc., Portland, OR, USA) was used to validate MS/MS-based peptide and protein identifications. Peptide identifications were accepted if they could be established at greater than 95.0% probability by the Scaffold Local FDR algorithm. Protein identifications were accepted if they could be established at greater than 99% probability and contained at least 1 identified peptide. Proteins that contained similar peptides and could not be differentiated based on MS/MS analysis alone were grouped to satisfy the principles of parsimony. Proteins sharing significant peptide evidence were grouped into clusters. 

A total of 1291 proteins were identified in Dataset 1 and in Dataset 2. Proteins recognized in less than four of the six samples were eliminated, yielding a Merged Dataset with 1216 proteins. We used PredAlgo (https://giavap-genomes.ibpc.fr/predalgo/) to predict the subcellular localization of each identified protein. We used SignalP 4.1 (www.cbs.dtu.dk/services/SignalP/) to identify proteins categorized as O (Other) or NA (Not Assigned) but predicted to contain a signal peptide (SP). The merged dataset contained 102 signal peptide-containing proteins, which we refer to as the soluble mating secretome. 

When possible, proteins in the soluble mating secretome were grouped on the basis of function, as assigned by Phytozome v12.1 (https://phytozome.jgi.doe.gov), Uniprot (https://uniprot.org), and literature searches. Where indicated, mammalian homologues of these proteins were identified using BLASTp (https://blast.ncbi.nlm.nih.gov) and the Uniprot database. NeuroPred (stagbeetle.animal.uiuc.edu/cgi-bin/neuropred.py) [5] and SMART (smart.embl-heidelberg.de) were used to predict cleavage sites and protein domains. For the analysis of proteases, and we utilized the hierarchical, structure-based classification system of Families and Clans available in the MEROPS database, Release 12.0 (https://www.ebi.ac.uk/merops/).

### 2.6. Bioinformatic Analyses

To search for any neuropeptide-like precursors encoded by the *C. reinhardtii* genome, we identified primary transcripts (Phytozome v5.5; a total of 17,741) encoding proteins with a predicted signal peptide (SignalP v4.1) and no transmembrane helices (TMHMM v2.0), resulting in a list of 771 proteins. Using a custom script, these protein sequences were searched for potential prohormone convertase cleavage sites ((K/R)X_n_(K/R), where *n* = 0, 2, 4 or 6); likely furin cleavage sites (RX(K/R)R) were screened for separately. The cleavage sites identified were then screened for the presence of potential amidation sites (G(K/R)(K/R), (K/R)X_n_G(K/R) where *n* = 1 or 3, and RG(K/R)R). In addition, NeuroPred [5] was used with “known motifs” to predict amidated product peptides that could be produced from these proteins; based on cleavages observed in *Aplysia californica*, RK, KXXK, and KXXR sites were excluded from “known motifs”. Multiple sequence alignments utilized T-COFFEE, Version 11.00 [25].

## 3. Results

### 3.1. The C. reinhardtii Genome Encodes Multiple Proteins with the Characteristics of Neuropeptide Precursors

**Figure 2:**
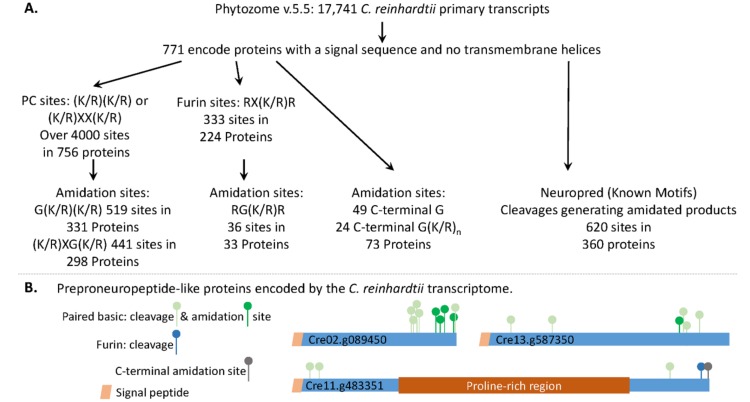
The *C. reinhardtii* transcriptome encodes multiple proteins that resemble classical neuropeptide precursors. (**A**) The strategies used to identify prohormone convertase (PC) and furin cleavage sites, and the amidated products that could be produced from signal peptide-containing soluble *C. reinhardtii* proteins, are outlined. Potential amidation sites that do not require the action of an endoprotease were also identified. The *C. reinhardtii* transcriptome was also analyzed using Neuropred (Known Motifs) [5] to identify potential amidated peptides and their lengths. (**B**) Diagrams illustrate the location of paired basic cleavage sites, furin sites, and amidation sites in three *C. reinhardtii* proteins that resemble classical neuropeptide precursors.

We screened the *C. reinhardtii* transcriptome for the presence of proteins that fit the generally accepted criteria for neuropeptide precursors. We limited our search to the 771 soluble proteins predicted to contain an N-terminal signal peptide (Figure 2A) (next page). Screening this set of proteins for consensus subtilisin-like prohormone convertase cleavage sites ((R/K)X_n_(R/K)↓, where n = 0 or 2, and ↓ identifies the cleavage site) [26], yielded 756 proteins. Potential amidation sites were identified in 331 proteins with G(K/R)(K/R) sites and in 298 proteins with (K/R)XG(K/R) sites. The prohormone convertases that recognize paired basic cleavage sites function optimally in the low pH environment of secretory granules; since furin does not require an acidic environment, we screened for furin-like cleavage sites (RX(K/R)R), which were found in 224 proteins; 33 could yield amidated peptides. Proteins with a C-terminal –Gly or –Gly–(Lys/Arg)_n_ can be amidated without prior endoproteolytic cleavage [27,28]; 49 of the 771 *C. reinhardtii* proteins were predicted to be secretory, terminate with –Gly, and 24 could be converted into PAM substrates by the action of a CPB-like enzyme. The full dataset is provided in Supplemental Table S1.

We also used Neuropred [5] to identify *C. reinhardtii* proteins that could generate amidated peptides following cleavages at “Known Motifs” (Figure 2A); 360 proteins were identified. A total of 620 amidated peptides, ranging in size from 2 to over 100 amino acids, were identified (Supplemental Table S2). While 60% of these proteins could yield only one amidated peptide, 30 could yield 3 amidated peptides and 4 could each yield 8 amidated peptides. All 20 amino acids were identified as potential products; Gly-amide, the predicted C-terminus of 94 of the amidated peptides, was the most prevalent.

Examples of precursors that could yield multiple amidated peptides, utilize furin and paired basic cleavage sites, and undergo C-terminal amidation, are shown in Figure 2B. The *C. reinhardtii* transcriptome encodes multiple proteins that could be acted upon by the classical neuropeptide processing machinery. 

### 3.2. The C. reinhardtii Genome Encodes Enzymes that Resemble the PCs and CBP-Like Enzymes Essential for Neuropeptide Production 

**Figure 3:**
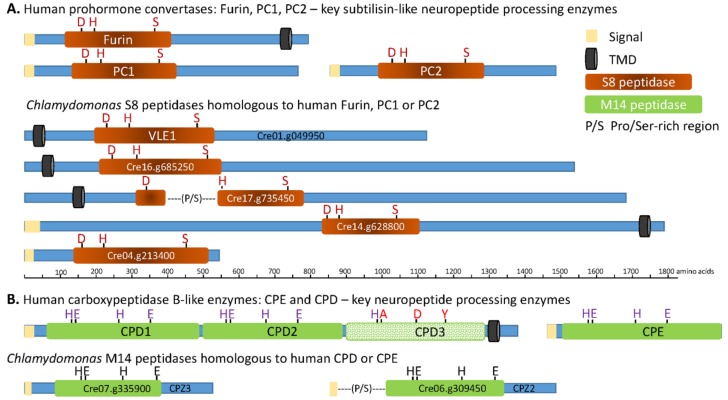
Subtilisin- and carboxypeptidase B-like proteins encoded by the *C. reinhardtii* transcriptome. The closest homologs of human PC1 (BAA11133.1), PC2 (AAB32656.1), furin (NP_002560.1), CPE (P16870.1), and CPD (AAH51702.1) were identified using NCBI BLASTp and v5.5 of the *C. reinhardtii* proteome (Phytozome 12). The subtilisin (S8) and CPB (M14) catalytic cores, signal peptide, transmembrane domains, and other features were identified using SMART and the MEROPS data base. Diagrams are drawn to scale, with active site residues indicated: D, H, and S form the catalytic *triad of S8 family proteases; the M14 peptidases rely on zinc binding to an H, E, H* motif, and an active site E. The third catalytic domain of CPD is not active; altered residues are shown in brown. P/S, indicates a Pro/Ser-rich region.

The first step unique to neuropeptide precursor processing involves limited endoproteolytic cleavage by subtilisin-like prohormone convertases as the newly synthesized proteins move through the secretory pathway lumen (Figure 1). When we searched for homologs of human furin, PC1, and PC2 in the *C. reinhardtii* genome using BLASTp, five subtilisin-like *C. reinhardtii* proteins were identified (Figure 3A). Like furin, Cre14.g628800 is a Type I integral membrane protein, while Cre04.g213400 is a soluble protein, as are PC1 and PC2. Strikingly, the other 3 homologs are predicted to be Type II integral membrane enzymes. Functional data are available only for Cre01.g049950, which encodes VLE1 (vegetative lytic enzyme, also known as sporangin), the endoprotease essential for the hatching of daughter cells from the mother cell wall [29].

The catalytic core (S8 domain) of subtilisin-like endoproteases includes an essential D, H, S catalytic triad; spacing of the active site residues in Cre01.g049950 (VLE1), Cre16.g685250, Cre14.g628800, and Cre04.g213400 resembles the spacing in furin, PC1, and PC2 (Figure 3 and Table 1). 

The catalytic cores of Cre01.g049950 (VLE1), Cre16.g685250, and Cre04.g213400 are most similar to those of human PC7, PC4, and PC1, respectively; the human enzymes each cleave secretory products after basic residues [13]. The catalytic core of Cre17.g735450, which is interrupted by a region rich in Pro and Ser (P/S), has short segments homologous to PC2 and PACE4, which also cleave after basic amino acids but, overall, is most homologous to a putative human subtilisin (SJM30502.1) that lacks a signal peptide. The catalytic core of Cre14.g628800 most closely resembles that of human SKI-1/S1P, which cleaves after non-basic sites in membrane-bound transcription factors [13].

Searching the Phytozome 12 (v5.5) database identified 21 *C. reinhardtii* proteins that contain the S8 peptidase domain (Table 1). SMART analysis predicts that 7 of the 21 proteins are secreted, 6 adopt a Type II topology, 1 has a Type I topology, and 7 may be cytosolic. A phylogenetic analysis identified a cluster containing the four *C. reinhardtii* S8 domain proteins most closely related to human furin, PC1, and PC2 (Supplemental Figure S2). The oxyanion asparagine in PC2 has been replaced by aspartic acid; this same substitution occurs in one of the 21 *C. reinhardtii* S8 domain proteins (Cre17.g713600). As expected, the S8 catalytic domain is well conserved, with evolutionary divergence reflected in features outside of the catalytic core [30]. 

To identify *C. reinhardtii* CPB homologs, we used the amino acid sequences of human CPE and CPD. Two CPB-like proteins were identified in *C. reinhardtii* (Figure 3B) (Supplementary Figure S3). Both contain a catalytic core (M14 peptidase; MEROPS database) that includes the three essential zinc-binding residues (H, E, H) and the active site Glu. The catalytic core of CPZ2 (Cre06.g309450) is preceded by a Pro/Ser-rich region. While human CPD is a Type 1 integral membrane protein, both CPZ2 (Cre06.g309450) and CPZ3 (Cre07.g335900) lack a transmembrane domain and, thus, resemble CPE.

### 3.3. Expression of Transcripts Encoding C. reinhardtii Neuropeptide Processing Enzymes Is Regulated During Sexual Reproduction

Neuropeptide processing enzyme expression often varies with neuropeptide expression [31,32,33]. Published transcriptomic data for *C. reinhardtii* reveal dramatic changes in expression of the *C. reinhardtii* genes that encode the proteases most closely related to human furin, PC1, PC2, CPD, and CPE [20] (Supplemental Figure S4). Transcript levels were reported for asynchronous vegetative cells and for cells synchronized using a light/dark cycle, resting gametes (*plus* and *minus*) and gametes of both mating types treated with lysin to remove the cell wall or with dibutyryl-cAMP to mimic flagellar activation of the mating signaling pathway. 

Expression of Cre04.g213400, the protein most homologous to PC1, was not reported in this dataset, but each of the four other subtilisin-like enzymes exhibits a unique pattern [20]. VLE1 (Cre01.g049950) is most highly expressed in vegetative cells. Expression of Cre16.g685250 is especially sensitive to lysin treatment, while expression of Cre17.g735450 increases in response to dibutyryl-cAMP, and expression of Cre14.g628800 drops in response to dibutyryl-cAMP. CPZ3 (Cre07.g335900), a CPB-like enzyme, is highly expressed in vegetative cells and resting gametes, dropping to low levels in lysin- or dibutyryl-cAMP-treated gametes (Supplemental Figure S4) [20]. By contrast, expression of CPZ2 (Cre06.g309450), another CPB-like enzyme, rises in response to Lysin-treatment and is higher after dibutyryl-cAMP treatment than in resting gametes. Different subtilisin-like and CPB-like *C. reinhardtii* enzymes appear to be used to perform distinct functions. The *C. reinhardtii* genome encodes a single PAM protein (Cre03.g152850) [16,34]; its expression drops after dibutyryl-cAMP-treatment.

### 3.4. Preparation and Analysis of the Soluble Mating Secretome

**Figure 4:**
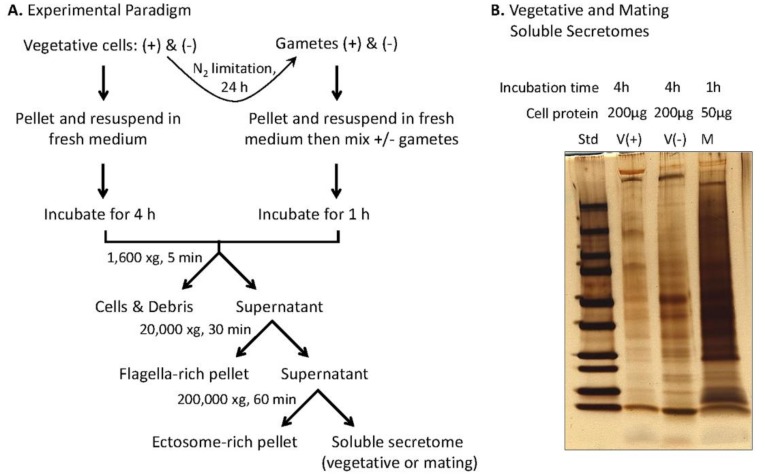
Preparation of the soluble mating secretome. (**A**) Experimental paradigm. Equal numbers of vegetative cells (*minus* and *plus*) (10–12 × 10^6^ cells/mL) suspended in fresh medium were grown in constant light for 4 h; the indicated series of centrifugation steps yielded the vegetative ectosome-rich pellet, and the soluble vegetative secretome. For preparation of mating ectosomes and the soluble mating secretome, gametes were prepared by incubation of vegetative cells in nitrogen-limited medium for 24 h; after mixing an equal number of *plus* and *minus* gametes (1–2 × 10^8^ cells of each mating type) in 10 mL nitrogen-free medium), mating was allowed to proceed for 1 h in constant light with gentle aeration. The mating medium was processed, as described, for the vegetative medium, yielding the mating ectosome enriched pellet and the soluble mating secretome. (**B**) SDS-PAGE analysis (4 to 15% polyacrylamide gradient gels) of the soluble vegetative secretome (*plus* and *minus*) and the soluble mating secretome. For vegetative cells, the volume of soluble secretome loaded came from 200 μg of cell protein; for mating cells, the volume of soluble secretome loaded came from 50 μg of cell protein.

In addition to vesicle-mediated secretion, *C. reinhardtii* release bioactive ectosomes from their cilia [35,36,37]; we used differential centrifugation to separate ectosomes from the soluble secretome. Vegetative cells and gametes of both mating types were prepared (Figure 4A). A series of differential centrifugation steps yielded an ectosome-rich pellet and the soluble secretome. The soluble vegetative secretome and the soluble mating secretome were visualized after SDS-PAGE (Figure 4B). The major proteins in the soluble vegetative secretomes varied with mating type. Furthermore, mating gametes released substantially more protein per unit time than vegetative cells. 

**Figure 5:**
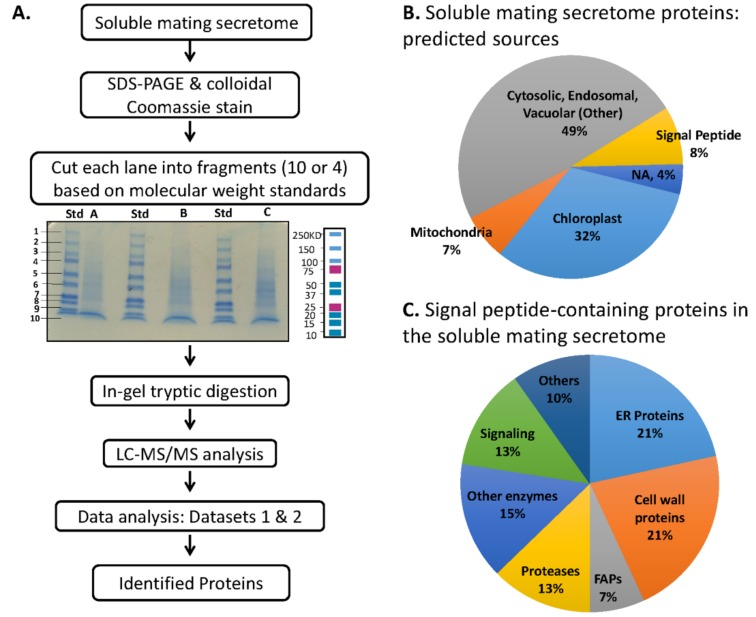
Analysis of soluble mating secretome. (**A**) Separate preparations of gametes were used on two different occasions to generate a total of six soluble mating secretome samples. For the first set of three samples, gels were sliced into 10 fragments, generating Dataset 1 (1233 proteins). For the second set of three samples, gels were sliced into 4 fragments, generating Dataset 2 (1494 proteins). The two datasets were combined as described in Materials and Methods, yielding a merged dataset; normalized spectral counts were used to calculate the spectral count average and standard error of the mean. Spectral count data, predicted location, signal peptide presence, number of transmembrane helices, functional information and data for individual gel slices appear in Supplemental Table S3. (**B**) Predalgo predictions for the subcellular localization of all proteins in the merged dataset were used to generate the pie chart. (**C**) For soluble mating secretome proteins predicted to contain a signal peptide, functional predictions were made using Phytozome and literature analyses (the complete list appears in Supplemental Table S4).

We fractionated soluble mating secretome samples by SDS-PAGE; a total of six independent samples were analyzed (Figure 5A). Proteins identified in at least 4 of the 6 samples are listed in Supplemental Table S3. Almost half were assigned to the category that includes cytosolic, endosomal and vacuolar proteins (Other), with 32% identified as chloroplast proteins (Figure 5B). The selective destruction of *minus* gamete chloroplast nucleoids and *plus* gamete mitochondrial DNA that accompanies sexual reproduction may contribute to the prevalence of proteins associated with organelles in the soluble mating secretome [21]. Signal peptide-containing proteins accounted for 8% of the secretome proteins; the complete list appears in Supplemental Table S4. ER and cell wall proteins each accounted for about one-fifth of the signal peptide-containing proteins, with proteases accounting for one-eighth (Figure 5C). 

ER chaperones (4) and cell wall proteins (12) were abundant, as expected. Further work will be required to determine whether the identification of Cre11.g477950, an ADP-ribosylglycohydrolase, as the most prevalent component of the secretome, indicates a prominent role for the reversible ADP-ribosylation of chaperone proteins in controlling protein folding and secretion in *C. reinhardtii* [38,39]. The prevalence of importin β, importin β-3 homolog, and Ran GTPase-activating protein in the secretome, is consistent with the suggestion that proteins involved in controlling nuclear pore traffic also play a role in controlling ciliary protein trafficking [40,41]. Three putative tRNA synthetases (Ala, Glu, Thr) were identified in the soluble mating secretome, with alanyl- and glutamyl-tRNA synthetase among the 30 most prevalent proteins (Table 2). These ancient enzymes, which catalyze the ATP-dependent attachment of a specific amino acid to its tRNA, are known to perform additional functions in other organisms, and several human tRNA synthetases are secreted [42,43,44]. 

Four of the seven *C. reinhardtii* proteins, identified as homologs of mammalian neuropeptide processing enzymes (Figure 3), were present in our analysis of mating medium that contained both ectosomes and soluble proteins. Only VLE1 (Cre01.g049950) was identified in the soluble mating secretome. VLE1 is released in vegetative ciliary ectosomes, which can cleave the mother cell wall [35]. It is not clear how VLE1 activation, which presumably involves an autoproteolytic cleavage, is triggered, or the subcellular site at which it occurs. It is possible that the other neuropeptide processing enzyme homologs identified (Cre16.g685250, Cre17.g735450 and Cre06.g309450 (CPZ2)), along with PAM (Cre03.g152850), will be found in mating ectosomes. 

### 3.5. Multiple Signal Peptide-Containing Proteases Are Present in the Soluble Mating Secretome

**Figure 6:**
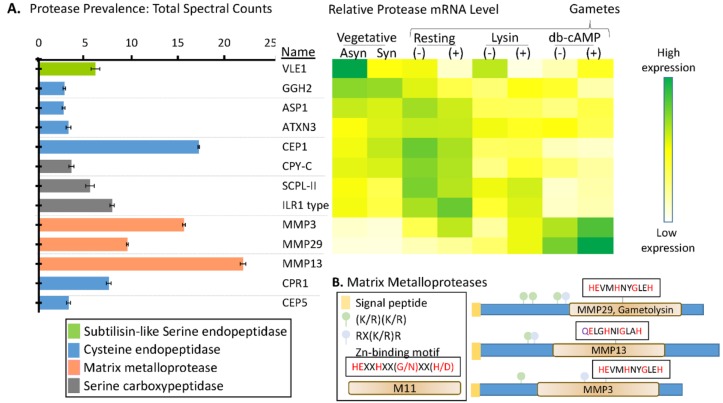
Signal peptide-containing proteases in the soluble mating secretome. (**A**) Average normalized total spectral counts (*n* = 6; SEM) are shown for each of the signal peptide-containing proteases identified in the merged dataset. The heatmap used transcriptomic data from Ning et al. [20] to identify stages during which mRNAs encoding many of these proteases are most highly expressed; transcriptomic data for MMP13, CPR1, and CEP5 were not available in the cited study. (**B**) Diagrams illustrating key features of the three MMPs identified. Furin recognition motif: RX(R/K)R; Zn-binding motif: HEXXHXX(G/N)XX(H/D) [47].

Proteases representative of several different classes were identified in the soluble mating secretome (Figure 6 and Supplemental Table S5). Cell wall removal during mating is accomplished by gametolysin, a zinc-containing matrix metalloprotease (MMP) [45,46]. Gametolysin is stored in the periplasm as an inactive 65 kDa proenzyme (progametolysin); its cleavage and activation are triggered by the increase in intracellular cAMP that occurs during mating [19,21]. A serine protease (p-lysinase) released in response to flagellar agglutination, cleaves and activates gametolysin [19]. With multiple candidate proteases, the genes encoding the proteases that account for these activities have not yet been identified. 

The only Ser endoprotease identified in the soluble mating secretome was VLE1, a type 2 integral membrane protein (Figure 6A). VLE1 is stored in cells as an inactive 127 kDa proenzyme; after hatching, active 125 kDa VLE1 can be recovered from the culture medium, presumably reflecting the occurrence of an endoproteolytic cleavage that separates active enzyme from the transmembrane domain [29]. Biochemical studies indicate some specificity of VLE1 for basic amino acids and two candidate sites (R^136^KR and R^168^R) precede the catalytic domain; the 9 tryptic peptides identified are consistent with autocatalytic cleavage at either of these sites. As expected, expression of VLE1 mRNA is highest in vegetative cells [20,29] (Figure 6). Lysin-catalyzed cell wall removal from *minus* gametes increases VLE1 mRNA levels, perhaps accounting for the presence of this protease in the soluble mating secretome. 

Three matrix metalloproteinases (MMPs), MMP3, MMP29, and MMP13, were identified. Based on spectral counts, the MMPs, which specialize in the degradation and remodeling of ECM and in the extracellular release of signaling proteins, were the most highly expressed proteases. Proteases more associated with lysosomal degradation (serine and metallo-carboxypeptidases and cysteine endoproteases) were also present. Expression of transcripts encoding MMP3 and MMP29 peak following db-cAMP treatment of both (*plus*) and (*minus*) gametes, consistent with a role for each of these enzymes in gametic cell wall removal. Expression of the remaining proteases peaked in vegetative cells or in resting gametes.

MMPs, ancient enzymes found in all kingdoms of life, are synthesized as inactive zymogens, with their signal peptide followed by a prodomain [47]. In plants, they are involved in remodeling the ECM and in cell–cell communication and signaling [48]. Each of the MMPs identified in the *C. reinhardtii* soluble mating secretome is predicted to be a soluble protein (Figure 6B). Based on homology to better characterized MMPs, activation will require an endoproteolytic cleavage that separates the prodomain from the catalytic domain. MMP activation is frequently catalyzed by subtilisin-like enzymes [49], and consensus furin-cleavage sites occur in the prodomains that precede the M11 catalytic domains in MMP29, MMP13, and MMP3 (Figure 6B). Based on transcript expression patterns, VLE1, Cre16.g685250, or Cre17.g735450 could be involved in activating MMPs (Supplemental Figure S3). 

The serine carboxypeptidases and cysteine and aspartyl endopeptidases identified are cathepsin-like, suggesting that they function in a degradative pathway. The human homologs of CEP1, CEP5, and CPR1 play a role in the degradation of extracellular matrix material. Transcripts encoding CEP1 and both serine carboxypeptidases (SCPL-II and CPY-C) are highest in resting gametes of both mating types, falling dramatically after lysin treatment or db-cAMP stimulation [20]. 

### 3.6. Many Cell Wall Pherophorins Recovered from the Soluble Mating Secretome Are Cleaved, While Hydroxyproline-Rich Proteins Remain Intact

Carefully controlled removal of the *C. reinhardtii* cell wall is required during vegetative growth, mating, and zygospore activation. Different sets of hydroxyproline-rich glycoproteins appear in the vegetative/gametic vs zygotic cell wall [21]. *Volvox* and *C. reinhardtii* cell wall proteins, termed pherophorins, resemble the subset of higher plant extensins referred to as solanaceous lectins, suggesting that their globular domains bind carbohydrates [50]. The pherophorins have a hydroxyproline-rich rod-like domain that separates globular N- and C-terminal domains [50]. Pherophorins are prevalent in the vegetative/gametic cell wall, but absent from early zygote-specific gene clusters [20,21]. The vegetative hatching enzyme (VLE1) must be specific enough to cleave the mother cell wall without attacking the vegetative cell wall. By contrast, the gametic lytic enzyme (gametolysin) can cleave the cell wall at all stages of the *C. reinhardtii* life cycle, except the zygospore [51]. Each of these proteases is produced as an inactive zymogen, with endoproteolytic removal of its prodomain, an essential part of the activation process. 

**Figure 7:**
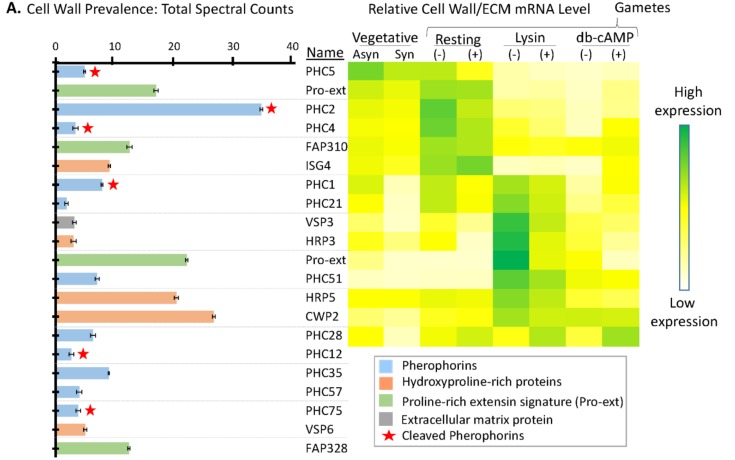
Signal peptide-containing cell wall proteins in the soluble mating secretome. (**A**) Average normalized total spectral counts are reported for cell wall proteins identified in the soluble mating secretome, as described in Figure 6. Cell wall proteins with a pherophorin domain were more prevalent than hydroxyproline-rich proteins. (**B**) As described in Figure 6, the heatmap uses transcriptomic data from Ning et al. [20]; PHC12, PHC35, PHC57, PHC75, VSP6, and FAP328 were not included in the cited analysis.

A total of 21 cell wall proteins were identified in the soluble mating secretome (Figure 7A). Three pherophorins were identified as highly expressed in vegetative cells (PHC5) or resting gametes (PHC2, PHC4) [20] (Figure 7B). Three pherophorins (PHC1, PHC21, and PHC51) and four hydroxyproline-rich proteins (VSP3, HRP3, HRP5, and CWP2), present in the soluble mating secretome, fell into the group of cell wall proteins whose transcript levels increased in response to g-lysin treatment of both *plus* and *minus* gametes [20,21]. Only two of the cell wall proteins identified (CWP2 and PHC28) fell into the group of transcripts whose levels responded to both g-lysin and dibutyryl-cAMP treatment of gametes. 

**Figure 8:**
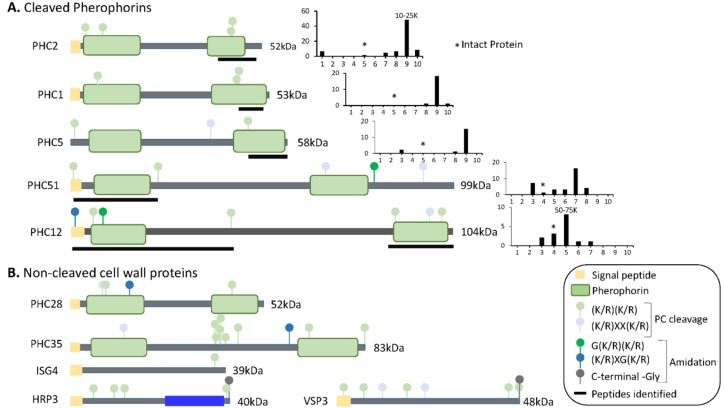
Predicted domain structures of cell wall proteins. (**A**) SMART was used to predict domain structures for cell wall proteins whose tryptic peptides were identified in gel slices containing proteins smaller than the intact protein. Signal peptides, pherophorin domains, and potential prohormone convertase cleavage sites are shown. To the right of each diagram, total spectral counts in each gel slice (from Dataset 1) are shown; * marks the gel slice in which that intact protein would be located; the mass range of the slice containing the highest spectral counts is indicated above the bar. Black bars under each diagram identify the region from which the tryptic peptides came. (**B**) The domain structures predicted for cell wall proteins, whose tryptic peptides were identified only in gel slices containing proteins at least as large as the intact protein, are shown.

In *Volvox*, cleavage of a cell wall protein releases a globular pherophorin domain that plays a role as a sexual inducer or pheromone [50]. By analyzing individual gel slices from both datasets (Supplemental Table S3), we looked for pherophorins or hydroxyproline-rich proteins that had undergone endoproteolytic cleavage, generating smaller stable products (Figure 8). 

For seven of the pherophorins, smaller fragments that contained peptides derived from one or both pherophorin domains were detected (Figure 8A). PHC2, the most abundant pherophorin, contains N- and C-terminal pherophorin domains. While intact PHC2 (52 kDa) was not detected, peptides from its C-terminal pherophorin domain were identified in the 10–18 kDa region of the gel. PHC1 and PHC5 also appeared to undergo cleavages that generated a stable C-terminal pherophorin domain (Figure 8A). Stable N- and C-terminal pherophorin domains were generated from PHC12. Seven of the eleven pherophorins identified in the soluble mating secretome had undergone cleavage; by contrast, the six hydroxyproline-rich cell wall proteins appeared to remain intact. While more detailed analyses will be required to identify the cleavage sites used, prohormone convertase-like cleavage of the pherophorins could generate the products observed (Figure 8A). 

## 4. Conclusions

**Figure 9:**
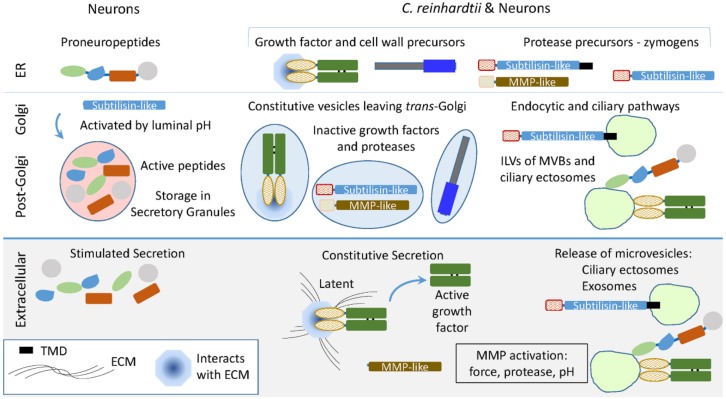
Using subtilisin- and MMP-like enzymes in *C. reinhardtii* and secretory granule-containing cells. The *C. reinhardtii* genome encodes subtilisin-like proteins resembling the enzymes that cleave proneuropeptides and generate the peptides stored in secretory granules (Table 1). MMP-like proteins resembling those that cleave latent (inactive) growth factor precursors extracellularly are also encoded by the *C. reinhardtii* genome. Ectosomes released from the cilia of hatching vegetative cells contain active VLE1, the subtilisin-like enzyme that degrades the mother cell wall. Latent growth factor activation in vertebrates involves extracellular activation of MMPs, along with the interaction of proteases and growth factors with the extracellular matrix and plasma membrane. The presence of secretory granules allows control of zymogen activation by declining luminal pH and storage of active peptides for release in response to secretagogues.

In neurons, the biosynthesis of neuropeptides, which are stored in secretory granules, and many growth factors, which are associated with the extracellular matrix, is orchestrated by the controlled cleavage of inactive precursors. Activation of the subtilisin-like endoproteases that produce most of the peptides stored in secretory granules is controlled by luminal pH, which declines as secretory products move from the ER, through the Golgi complex, and into secretory granules (Figure 9). Although secretory granules have not yet been identified in *C. reinhardtii*, their transcriptome encodes candidate preproneuropeptides. VLE1 (sporangin), a subtilisin-like enzyme resembling furin, PC1, and PC2, plays an essential role in vegetative growth, while gametolysin, an MMP-like enzyme resembling those responsible for the extracellular cleavage of proTGFβ family members, is essential for sexual reproduction. 

In addition to constitutive secretion of vesicles exiting the *trans*-Golgi, *C. reinhardtii* use ciliary ectosomes to deliver essential cargo to the appropriate target. The endomembrane system is well developed in *C. reinhardtii*, with multivesicular bodies (MVBs), contractile vacuoles, and acidocalcisomes. Topologically, the formation of ciliary ectosomes resembles the formation of intraluminal vesicles in MVBs. Upon MVB fusion with the plasma membrane, exosomes are released. Vertebrate PAM and furin function in the endocytic pathway, with PAM identified in exosomes from several different sources [52,53]. Our data suggest that the controlled endoproteolytic activation of proneuropeptides and growth factors had their molecular and enzymatic origins in unicellular organisms. The complex endomembrane system thought to be present in the last eukaryotic common ancestor presumably supported the evolution of the preproneuropeptides and growth factors essential for nervous system development and adult nervous system function well before the appearance of neurons.

## Figures and Tables

**Table 1:** S8 domain-containing subtilisin-like proteins in the *C. reinhardtii* genome.

Protein ID	Length	Signal	TM Helix	S8 Location	Most Closely Related Human Protein (*GENE*)
**Secreted**					
**Cre02.g076950**	1355	1–22	no	189–992	
**Cre04.g213400**	539	1–23	no	171–521	PC1 (*PCSK1*)
**Cre07.g329500**	945	1–29	no	601–847	
**Cre10.g459450**	866	1–30	no	536–836	
**Cre05.g242100**	1264	1–26	no	428–991	
**Cre05.g242750**	1301	1–23	no	511–1053	
**Cre19.g750447**	1141	1–26	no	700–1140	
**Type I membrane**					
**Cre14.g628800**	1787	1–47	1719–1741	837–1103	SKI-1 (*MBTPS1*)
**Type II membrane**					
**Cre01.g049950 VLE1, sporangin**	1117	no	37–59	210–539	PC7 (*PCSK7*)
**Cre03.g145827**	1512	no	45–67	219–552	
**Cre16.g685250**	1532	no	55–77	233–562	PC4 (*PCSK4*)
**Cre17.g708400**	1794	no	451–473	767–1163	
**Cre17.g735450**	1674	no	138–160	333–774	PC2 (*PCSK2*) & PACE4 (*PCSK6*)
**Cre03.g190250**	1229	no	1149–1171	298–792	
**Other**					
**Cre13.g585800**	809	no	no	154–425	
**Cre16.g675350**	1492	no	no	119–599	
**Cre17.g713600**	1982	no	no	764–1116	
**Cre03.g170300**	1374	no	no	Split	
**Cre05.g242700**	777	no	no	26–383	
**Cre05.g242856**	1419	no	no	585–1129	
**Cre09.g406700**	1890	no	no	482–1018	

For each S8 domain-containing protein, SMART was used to determine its total number of amino acids, the presence and length of any signal peptide (Signal), the location of potential transmembrane helices (TM Helix), and the position of the S8 domain (S8 location). Proteins highlighted in gray were identified based on screening for homologs to full-length human furin, PC1, and PC2. Based solely on their S8 domains, the most closely related hPCSK is identified; the preferred protein name is shown, with the corresponding gene name in parenthesis. Cre01.g0499450 was previously identified as sporangin (also known as vegetative lytic enzyme (VLE1)) [29].

**Table 2:** Thirty most prevalent components of the soluble mating secretome.

Rank	Accession Number	Mol Mass	Avg Tot Spectral Counts	SEM/ Avg	Description
**1**	Cre11.g477950.t1.1	94 kDa	189.1	0.13	ADP-ribosylglycohydrolase
**2**	Cre02.g088200.t1.2	58 kDa	148.4	0.09	Protein disulfide isomerase 1, RB60
**3**	Cre02.g143200.t1.1	122 kDa	71.7	0.05	Alanine tRNA ligase
**4**	Cre02.g080700.t1.2	72 kDa	66.9	0.10	ER associated Hsp70 protein
**5**	Cre01.g038400.t1.2	47 kDa	56.4	0.15	Calreticulin 2, calcium-binding protein
**6**	Cre14.g633750.t1.1	122 kDa	51.7	0.07	Importin β-3 homolog
**7**	Cre06.g298650.t1.2	53 kDa	51.0	0.43	Translation initiation factor 4A
**8**	Cre02.g080650.t1.2	93 kDa	41.4	0.09	ER associated heat shock protein 90B
**9**	Cre09.g394200.t1.1	156 kDa	37.7	0.34	Flagellar associated protein
**10**	Cre01.g034000.t1.2	97 kDa	37.3	0.26	Importin β
**11**	Cre14.g620600.t1.2	52 kDa	34.7	0.24	Pherophorin, PHC2
**12**	Cre09.g406600.t1.1	38 kDa	33.7	0.35	ChlamyFPv5, 2 KCl peptides
**13**	Cre06.g258800.t1.1	132 kDa	26.7	0.24	OH-Pro-rich glycoprotein, GP2 (FAP3)
**14**	Cre10.g431800.t1.2	70 kDa	25.2	0.37	Arylsulfatase
**15**	Cre07.g330200.t1.2	30 kDa	23.2	0.12	Radial spoke protein 9
**16**	Cre02.g089500.t1.2	43 kDa	22.1	0.21	Proline rich extensin signature
**17**	Cre03.g144564.t1.1	81 kDa	22.0	0.30	Matrix metalloproteinase, MMP13
**18**	Cre07.g321400.t1.1	199 kDa	20.5	0.13	Flagellar associated protein
**19**	Cre02.g089450.t1.2	38 kDa	20.4	0.33	Proline rich extensin signature, HRP5
**20**	Cre02.g077850.t1.2	83 kDa	18.6	0.34	Flagellar associated protein, FAP212
**21**	Cre09.g407700.t1.2	54 kDa	17.3	0.08	Cysteine endopeptidase, CEP1
**22**	Cre09.g401900.t1.2	132 kDa	16.9	0.37	Proline rich extensin signature
**23**	Cre12.g487700.t1.2	77 kDa	15.9	0.13	Serine/threonine protein kinase
**24**	Cre09.g393700.t1.1	71 kDa	15.7	0.16	Matrix metalloproteinase, MMP3
**25**	Cre11.g467547.t1.1	83 kDa	13.0	0.26	Glutamyl/glutaminyl-tRNA synthetase
**26**	Cre02.g077800.t1.2	86 kDa	12.5	0.45	Proline rich extensin signature (FAP310)
**27**	Cre02.g102050.t1.1	92 kDa	12.4	0.20	Proline rich extensin signature (FAP328)
**28**	Cre11.g479250.t1.2	54 kDa	12.1	0.11	Ran GTPase-activating protein,
**29**	Cre06.g304500.t1.2	40 kDa	10.9	0.18	Zygote-specific protein
**30**	Cre12.g533100.t1.1	21 kDa	10.6	0.41	CHRD domain, PF07452

The signal peptide-containing proteins identified in the merged dataset were sorted by average total spectral counts (Avg Tot Spectral Counts); the entire list, grouped by function, is provided in Supplemental Table S4. The 30 most prevalent proteins, which account for 72% of the total spectral counts, are shown here.

## Supplementary Materials

The following are available online at http://www.mdpi.com/2227-7382/6/4/36/s1, Figure S1: Alignment of five S8 subtilisin-like *C. reinhardtii* proteins with human homologs; Figure S2: Alignment of S8 domains of 21 *C. reinhardtii* family members; Figure S3: Alignment of two M14 CPB-like *C. reinhardtii* proteins with human proteins homologs; Figure S4: Expression of *C. reinhardtii* subtilisin-like, CPB-like and PAM transcripts; Table S1: Preproneuropeptides encoded by the *C. reinhardtii* transcriptome; Table S2: Neuropred analysis of *C. reinhardtii* transcriptome; Table S3: Mass spectrometric analysis of soluble mating secretome proteins. SolMatSecretome MergedDatasets; Table S4: Signal peptide-containing proteins; Table S5: Proteases identified in the soluble mating secretome.
