# Supplementary material for: Proteases Shape the Chlamydomonas Secretome: Comparison to Classical Neuropeptide Processing Machinery
Source: Proteomes. 2018 Sep 23;6(4):36. doi: 10.3390/proteomes6040036 (PMC6313938; doi:10.3390/proteomes6040036)
Supplement: Supplementary file 1 [file proteomes-06-00036-s001.zip › SupplementalFigures.pdf]

Supplemental Fig.1. Alignment of five S8 subtilisin-like *C. reinhardtii* and human proteins.

MSA *The multiple sequence alignment result as produced by T-coffee.*

```
T-COFFEE, Version_11.00.8cbe486 (2014-08-12 22:05:29 - Revision 8cbe486 - Build 477)
Cedric Notredame; SCORE=62. *BAD AVG GOOD* TCS (transitive consistency score) color code
goes from 0 (blue) to 9 (red).

                                                                        &
Fur            RDVYQEPTDPKFPQQWYLSGVTQR------DLNVKAAWAQ--------GYTGHGIVVSILDDGIEKNHPDLA
PC1            DSALNLFNDPMWNQQWYLQDTRMTAALPKLDLHVIPVWQK--------GITGKGVVITVLDDGLEWNHTDIY
PC2            NEIDINMNDPLFTKQWYLINTGQADGTPGLDLNVAEAWEL--------GYTGKGVTIGIMDDGIDYLHPDLA
Cre01.g049950  AYT---LNDPQLSTTWWFDR-----------INVTGAWRLLNVNATDPQQRTSDVVIGVTDSGAFVNHPDLI
Cre17.g735450  GGGGAVAADPLQASQWHLDA-----------VGAAEAWRV--------TSGVREVIVAVLDTGVDLAHPDLA
Cre16.g685250  QSVTVVPGAPDPLDQYSLGI-----------VKAKDAWNV--------TQGTPEVIVAVVDTGCDLTHPDLK
Cre04.g213400  GPITSNPTDDLWDQQWAPRA-----------IKAQSAWAA--------GVTGKCVRVAVLDTGMWWDHPDLA
Cre14.g628800  AGGGTVP----------V-M-----------LEADKLWKL--------GYTGRGVRVGIFDTGIRPDHPHIR
cons                                          : .   *                : : : * *    *..:


Fur            GNYDPG--------------------AS-FDVNDQDP-----------------------------------
PC1            ANYDPE--------------------AS-YDFNDNDH-----------------------------------
PC2            SNYNAE--------------------AS-YDFSSNDP-----------------------------------
Cre01.g049950  GSFWENPAEIDNDLRDNDNNTYIDDVYG-ACFANSVCSPT--------------------------------
Cre17.g735450  PNLWVNTGEVPNNGIDDDGNGFVDDAFG-YDFAGPCSTDWRAVPPPPPSPPPRPPAPPSPPPSPPPPPSPPP
Cre16.g685250  DNLWINTKEIPGNGVDDDGNGLIDDVYG-YDFAGTCENDY--------------------------------
Cre04.g213400  GRVDVD--------------------YA-RSFVDG-------------------------------------
Cre14.g628800  HI------------------------RERTNWTHQ-------------------------------------
cons

Cre17.g735450  RPRPPSPPPPPKRAPPPPARKTSGRRLVSGAAATHGGRRLLSALLGSVWGAGSSEALEATESLQHAQQAATA

                                                                             &
Fur            ------------------------------------D-PQ------PRYTQM-N--D--NRHGTRCAGEVAA
PC1            ------------------------------------D-PF------PRYDPT-N--E--NKHGTRCAGEIAM
PC2            ------------------------------------Y-PY------PRYT---DDWF--NSHGTRCAGEVSA
Cre01.g049950  ------------------------------SLNSNLSRCGIGKNTFAWNNVN-D--I--NSHGTKIAGVIGA
Cre17.g735450  AAAATKEGASGGSSSSQGQGRRRLLDSAPVDQVYLPGRCG------SDPDPRPE--SGDTGHGTHVAGIIGA
Cre16.g685250  ----------------------------------PSDGCG------PKPDPQ-D--T--GTHGSHCAGIVGA
Cre04.g213400  --------------------------------------IP------PYADSQ-N--Y--FWHASHVAGIVAG
Cre14.g628800  --------------------------------------Q----------SLS-D--G--LGHGTFVAGVVGG
cons                                                                :       *.:  **  :.
```

```
Fur            VANNGVCGVGVAYNARIGGVRMLDGE-----VTDAVEARSLGL-----NPNHIHIYSASWGPED-DGKTVDG
PC1            QANNHKCGVGVAYNSKVGGIRMLDGI-----VTDAIEASSIGF-----NPGHVDIYSASWGPND-DGKTVEG
PC2            AANNNICGVGVAYNSKVAGIRMLDQPF----MTDIIEASSISH-----MPQLIDIYSASWGPTD-NGKTVDG
Cre01.g049950  RPNNGIGLAGVAPNLRQMVLKVVDDTYFQYAYSDVVRAIDYGY------AKGARIFSMSFGQDARTSATPTN
Cre17.g735450  VRGNGVGGSGIAPGVRVMPLKVSDPQGNFW-ASNIVAAYDYAH------RMGAHVVSCSFGPYQ-PTLNPDA
Cre16.g685250  VRNNGVGISGIAPRVKIMCLKVSTPSNNFY-TAHILKAYDYAY------RMGAHVVSCSFGPAE-PNMKTIS
Cre04.g213400  SANNG-GIVGIAPEATIIPIKVLDNNAG-D-NSFLIQGLLYAASPLGPDGAGADIINLSLGGFGNPNDKN--
Cre14.g628800  QD--G-ACPGFAPDVEMLTFKVFTDDQVSY-TSWFLDAFNYAI------VVKVNVINLSIGGP-D--Y----
cons                    *.*       .::          :  : .   .            : . * *
```

$ (oxyanion residue)

```
Fur            -PARL----AEEAFFRGVSQGRGGLGSIFVWASGNGGREHDSCNCDGYT--NSI-------YTLSISSATQF
PC1            -PGRL----AQKAFEYGVKQGRQGKGSIFVWASGNGGRQGDNCDCDGYT--DSI-------YTISISSASQQ
PC2            -PREL----TLQAMADGVNKGRGGKGSIYVWASGDGG-SYDDCNCDGYA--SSM-------WTISINSAIND
Cre01.g049950  KPSLD----AAATAYRNLFN--KYSNALFVAAAGNEWTSLEGWRSGNYTYSPCMIA---TDNTLCVGGTN-V
Cre17.g735450  -I-DLAQAAAETRLYERAVRPLADKGVLLVAAAGNEDTNLDRARAVGMPYLPCTLD---LPNVLCVAASN-T
Cre16.g685250  -PDELAAYVAERNLYRAALQPMVDKNMLIIAAAGNENTNLDQLDQYNTTYMPCFMAKEFPENMLCVTATN-Q
Cre04.g213400  ---------DTWALYNKVLNYVASQGVLVVAAAGNDALNLGRTR--NTFVSPCEN----GGNTVCVSSTG-P
Cre14.g628800  ---------LDQPFVDKVLE-ITSNGVLLVSAIGNDGPLYGTLN--NP----A-D----QNDVIGVGGID-N
cons                              .     . : : * *:           .     .          : : .
```

```
Fur            GNVPWY-----------------SEACS----STLA-TTYSSGNQ--NEKQIVTTDL---------------
PC1            GLSPWY-----------------AEKCS----STLA-TSYSSGDY--TDQRITSADV---------------
PC2            GRTALY-----------------DESCS----STLA-STFSNGRKRNPEAGVATTDL---------------
Cre01.g049950  NDTIFY-----------------VFAFNQQAGTNFGPTTVDM---GAPAHSIYTTDI---------------
Cre17.g735450  QGGIWR-----------------EIVANRWVGSNTGPTTVDL---AAPGSRIVSTLP---------------
Cre16.g685250  DDVRWA-----------------EIANNKPVGSNFGLKYADI---GAPGRLIMSSTP---------------
Cre04.g213400  LDVCKTCQDD-WINTCLESPSTWDLKQQLALYSNYGPSAISV---AGPGGDYRSAFEWYAKWYDPATDSFLP
Cre14.g628800  WDNI---ASFSSR-----GMSTW------ELPRGYGRVKPDV---MAYAKDVQG------------------
cons                                              .    .
```

&

```
Fur            -----------------R--QK---CTESHTGTSASAPLAAGIIALTLEANKN-------------------
PC1            -----------------D--ND---CTETHTGTSASAPLAAGIFALALEANPN-------------------
PC2            -----------------Y--GN---CTLRHSGTSAAAPEAAGVFALALEANLG-------------------
Cre01.g049950  -----------------A-VNR---NYSAPSGTSFATPMVAAVAGLVLSALGG--------------TGRA-
Cre17.g735450  -----------------G-AT-----YGDKTGSSMATPLVSGSAALVLSALGAVDGNY--------------
Cre16.g685250  ---PV------------A-GNSMT-RWNLKTGSSMATPMVSGIGALVLSVLGTGTGNY--------------
Cre04.g213400  GTPPTCWFVDQVLSVGAPNRPDLP-AFIQYTGTSQAAPAVSGVAALMIHDAAVRQGVDLCAASRAGLKGRAR
Cre14.g628800  --------------------SKITGGCRSLSGTSVASPVVAGAVSLLASTVLP-------DKPGAGEDGRW-
cons                                         :*:* ::* .:.  .*
```

```
Fur              --LTWRDMQHLVVQTSK--P--A
PC1              --LTWRDMQHLVVWTSEYDP--L
PC2              --LTWRDMQHLTVLTSK--RN-Q
Cre01.g049950    TPQTPLQIKNILMSS--GDL--L
Cre17.g735450    --FQAVRARSLLLEG--GDP--Q
Cre16.g685250    --FQGKEARKIIVES--A----D
Cre04.g213400    GPLNVAQIKTALARG--AV-YPG
Cre14.g628800    ALLNPGSMKQALVEG--AVRLPN
cons                     :
```

Supplemental Fig.1. Alignment of five S8 subtilisin-like *C. reinhardtii* proteins with human homologs. The *C. reinhardtii* proteins most homologous to human furin, PC1 and PC2 were identified using BLASTp. Their catalytic core sequences were then aligned using T-Coffee. The residues that form the catalytic triad (D, H and S) are marked in red. The oxyanion residue (N in all prohormone convertases except for PC2, where it is D) is also marked in red. In Cre17.g735450, an insert containing a Pro-rich region separates catalytic triad residues D and H.

Supplemental Fig. 2. Alignment of *C. reinhardtii* S8 domain proteins. The phylogenetic tree was constructed using Clustal Omega with neighbor end joining. Multiple sequence alignments for each Cluster were done using T-COFFEE, Version_11.00. Catalytic triad members (D, H and S, &) and the oxyanion residue ($) are marked.

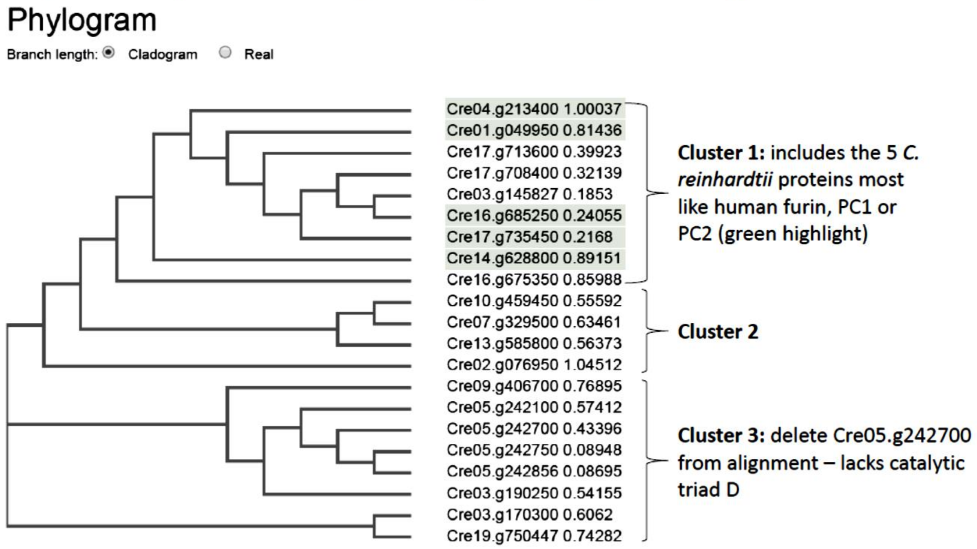

T-COFFEE, Version_11.00.d625267 (2016-01-11 15:25:41 - Revision d625267 - Build 507)Cedric Notredame SCORE=68
BAD AVG GOOD TCS (transitive consistency score) color code goes from 0 (blue) to 9 (red).

Cluster 1: Cre01.g049950 (VLE1, sporangin); Cre03.g145827; Cre04.g213400; Cre14.g628800; Cre16.g685250; Cre17.g708400 (Pro-rich insert between H and oxyanion residue); Cre17.g713600 (Pro-rich insert between H and oxyanion residue and N → D for oxyanion residue); Cre17.g735450 (Pro-rich insert between H and oxyanion residue).

```
                         &                                                                                         &
Cre01.g049950   TSDVVIGVTDSGAFVNHPDLIG----SFWENPAEIDNDLRDNDNNTYIDDVYGACFANS--VCS-PTSLNS------------------NNVNDINSHGTKI
Cre03.g145827   VSDVVVAVIDTGCDLTHPDLQG----QLWTNPGEIPGNGVDDDGNGYIDDVHGFDFAGS---CE-SDWRSTK-----------------PDPQDTHSHGTHC
Cre04.g213400   GKCVRVAVLDTGMWWDHPDLAG----RVDVDYARSFVD-----------GI--------------------------------------ADSQNYFWHASHV
Cre14.g628800   GRGVRVGIFDTGIRPDHPHIRHIRERTNWTHQQSL--------------------------------------------------------SDGLGHGTFV
Cre16.g685250   -PEVIVAVVDTGCDLTHPDLKD----NLWINTKEIPGNGVDDDGNGLIDDVYGYDFAGT---CE-NDYPS-------------------PDPQDTGTHGSHC
Cre17.g708400   P-DVIVAVVDTGFDIDHPDLKP----NLWVNPGEVPGDGIDNDGNGFIDDINGFDFAGSGDACQ-GDWRARA-(53 aa insert)--PESSD-TGHGTHV
Cre17.g713600   --TVVVAVVDGGFDTRHPDLAG----ALWVNPGEIPGNGIDDDGNGFVDDVNGWDFGGN---CSSGAWRPAP-(43 aa insert)--PEAGD-GGHGTHV
Cre17.g735450   VREVIVAVLDTGVDLAHPDLAP----NLWVNTGEVPNNGIDDDGNGFVDDAFGYDFAGP---CS-TDWRAVP-(133 aa insert)-PESGD-TGHGTHV
cons               * :.: * *    **.:          .                                                              :   *.:
```

```
Cre01.g049950   AGVIGARPNNGIGLAGVAPNLRQMVLKVVDDTYFQYAYSDVVRAIDYG------YAKGARIFSMSFGQD-ARTSA--TPTNKPSLDAAATAYRNLFNK--YS
Cre03.g145827   SGIVAALRN-----IGIAPRVKVMCLKVATPNGAFYT-SHILKAYDYA------YNMGAHIASCSFGPA-EPNLQP-TPQEVEEMWNETKFYKTALDPMVKK
Cre04.g213400   AGIVAGSANNG-GIVGIAPEATIIPIKVLDNNAG-DN-SFLIQGLLYAASPLGPDGAGADIINLSLGGFGNPNDK-ND---------TWALYNKVLNYVASQ
Cre14.g628800   AGVVGGQD--G-ACPGFAPDVEMLTFKVFTDDQVSYT-SWFLDAFNYA------IVVKVNVINLSIGGP-DYLDQ---P-----------FVDKVL-EITSN
Cre16.g685250   AGIVGAVRNNGVGISGIAPRVKIMCLKVSTPSNNFYT-AHILKAYDYA------YRMGAHVVSCSFGPA-EPNMKTISPDELAAYVAERNLYRAALQPMVDK
Cre17.g708400   AGIVAAVRNSVAAVAGAAPRVKVMVLKVFDASGRVYA-SHVVAAYAYA------QRMGAHIVSCSFGPD-TPNLQ-PQPYEVAEMVAQESLYSSAVTPLESK
Cre17.g713600   AGVIAAVRKDLAGTSGVAPHVRLMLLKVVDGYGATYG-SRVAAALEYA------GRMGAHVVVTSVGPS-APLPSAPRPYQLAANAAAAVYAAAVGSLRDK
Cre17.g735450   AGIIGAVRGNGVGGSGIAPGVRVMPLKVSDPQGNFWA-SNIVAAYDYA------HRMGAHVVSCSFGPY-QPTLN-PDAIDLAQAAAETRLYERAVRPLADK
cons            :*::..         * **    : :**          : .  .  *.          . :   *.*                            .     .

                                  $ (oxyanion residue)
Cre01.g049950   NALFVAAAGNEWTSLEGWRSGNYTYSPCMIA-----TDNTLCVGGTNVNDTIFY----------------VFAFN---QQAGTNFGPTTVDMGAPAHSIYT
Cre03.g145827   GMLVVAAAGNENTPLESLDAVNSTYNPCFMAR--YFADSMLCVMATNPNDSRWF----------------EIAAN---KPIGSNYGTKYADIGAPGRQIYS
Cre04.g213400   GVLVVAAAGNDALNLGRT--RNTFVSPCEN------GGNTVCVSSTGPLDVCKTCQDDWINTCLESPSTWDLKQQ---LALYSNYGPSAISVAGPGGDYRS
Cre14.g628800   GVLLVSAIGNDGPLYGTLN------NPAD-------QNDVIGVGGIDNWDNIAS----------------FSSRGMSTWELPRGYGRVKPDVMAYAKDVQG
Cre16.g685250   NMLIIAAAGNENTNLDQLDQYNTTYMPCFMAK--EFPENMLCVTATNQDDVRWA----------------EIANN---KPVGSNFGLKYADIGAPGRLIMS
Cre17.g708400   GVLLVAAAGNELTDLNGLASVKSNYLPCTLP-----NSNVMCITGSNPLDRAIT----------------GMAGN---MPVGVNYGTAVVDMAAPGQDIYN
Cre17.g713600   GVLVVAPAGDDGIDLDAAKAAGLEYLPCTLAAPPYSLGNVLCVGAADASDRRIRI---------------AVPGLD---PALGSNYGSSSVSIAAPGVDIYS
Cre17.g735450   GVLLVAAAGNEDTNLDRARAVGMPYLPCTLD-----LPNVLCVAASNTQGGIWR----------------EIVAN---RWVGSNTGPTTVDLAAPGSRIVS
cons            . *.::. *::               *.          : : : . .  .                                 . *   .:  . .

                                                   &
Cre01.g049950   TDI---------AVN---------------------------RNYSAPSGTSFATPMVAAVAGLVLSAL--------------GGTG--
Cre03.g145827   TVP---------KLI----GN-------------------GYATYAYKTGSSMATPMTAGVGALVLSVL--------------GAG---
Cre04.g213400   AFEW-YAKWYDPATDSFLPGTPPTCWFVDQVLSVGAPNRPDLPAFIQYTGTSQAAPAVSGVAALMIHDAAVRQGVDLCAASRAGLKGR-
Cre14.g628800   SKI---------T-----------------------------GGCRSLSGTSVASPVVAGAVSLLASTVLPD-------KPGAGEDG--
Cre16.g685250   STP---------PVA----GN-------------------SMTRWNLKTGSSMATPMVSGIGALVLSVL--------------GTG---
Cre17.g708400   TFP---------ISL---------------------------GSYGNKTGSSMATPLVAGVAALVASVV--------------GAVGPL
Cre17.g713600   TLPDRWAQVVDPVTG---------------------------GGYGNMSGSSAAAPVVAGVAALVVGV---------------------
Cre17.g735450   TLP---------G-----------------------------ATYGDKTGSSMATPLVSGSAALVLSAL--------------GAV---
cons            :                                               :*:* *:* .:.  .*:

Cre01.g049950   --RAT-PQTPLQIKNILMSSGDLLPSLNN--QFKSARRL----------
Cre03.g145827   --TGN-YFQGQAARKIIVESADRLVDLPV--R--TGSRINAQRAVQQAR
Cre04.g213400   ---ARGPLNVAQIKTALARGAV-YPGGAVDREAYGA------------G
Cre14.g628800   --RWA-LLNPGSMKQALVEGAVRLPNLNI--YEQGS------------G
Cre16.g685250   --TGN-YFQGKEARKI---------------------------------
Cre17.g708400   VASPN-YYQASSVKSLLLETADKLPGLPV--I--GGRRLNAQRAVEA-A
Cre17.g713600   -------------------------------------------------
Cre17.g735450   --DGN-YFQAVRARSLLL------------------------------E
cons
==================================================================================================
```

MSA *The multiple sequence alignment result as produced by T-coffee.*

```
T-COFFEE, Version_11.00.d625267 (2016-01-11 15:25:41 - Revision d625267 - Build 507) Cedric Notredame SCORE=64
*BAD AVG GOOD* (transitive consistency score) color code goes from 0 (blue) to 9 (red).
```

Cluster 2: Cre02.g076950 (Insert between oxyanion residue and S); Cre07.g329500; Cre10.g459450; Cre13.g585800; Cre16.g675350 (Insert between D and H).

```
                         &
Cre02.g076950   GWDVKVGIVDTGVDYRHPALG------G-------------------------------GFGPG------------------FKVAYGR-----------DF-
Cre07.g329500   GSGSHIYVLDTGIRSTHYAFS------G-------------------------------------------------------RVGAGT-----------SM-
Cre10.g459450   -AGVTIYALDSGVFAQHDEFQ------SW---------GTAPA-----------AG--SGAATA------------------GRASYGH-----------DF-
Cre13.g585800   -TGVTAYVVDSGVRGSHTQFR------PL----------LPPA-----------QQ--PAAPTA---------PRAVSPSGPSRVQHGY-----------SVF
Cre16.g675350   GRGVKICIFDTGVDPGAAGLQITTDGKPK-(25 aa)-CIAGASGRKLRLNPDWKNPTGEWRVG-(56 aa)-KKERAELE-GRVAALK -(58 aa)--GTF
cons              .     .*:*:      :                                                               :.               .

                                              &
Cre02.g076950   AGDA---FKPGGVPA-----PDADPMDCAGHGTHVAGIIAGSYSSADWSFSGVAPGVTLGAYKVFGCTG--GTTSELVVAALDAA----AADGMQ--VINLSL
Cre07.g329500   --------------------LGSSWEDDHGHGTHVSGTAMGAT-------YGVARAAIVHAVKVCDSQG--SGTYSNFISGLGWVKNHVQNNGIKQAVVSMSL
Cre10.g459450   VD------------------GDAEAADCDGHGTHVASTAVGRS-------VGVARGAELVAVRVLDCSG--SGSIADTVAGLDWL----AKHVKRPAVAMLSL
Cre13.g585800   TATA---GG---LGG-----SGGGDSDCYGHGTHVASLLGGVD-------YGVAKNVTIVPVQVLDCNG--NTSETLLKQGLEWV----GRNLVLPAVVHMSL
Cre16.g675350   SELDACNFV---LNILDGGRTLSVVVDCGAHGTHVAGITAAHFPD-DPGSNGIAPGAQIISCKIGDTRLGSMETGTGVVRGLIAA----RQH--GAHLINMSY
cons                                  .   *  .*****:.   .          *:*  . : . :: .       :      .*         .     :  :*

                                                    $ (oxyanion residue)
Cre02.g076950   GD-EGAWG----GPVAEAAARLA-G-LGVLVVAAVGNAGP-SG-(231 aa insert)-NALTPTPVPA--TIPALSPQPSPPPSPGQGQGQTGGNGATQGS
Cre07.g329500   ---SGPRS----ASLNDAIQDVV-N-NGITVVVAAGNNNGGDS-----------------CSYSPGSAPA--AISV---------------------------
Cre10.g459450   GVPAGDWS----RVLGEAVSVLV-SQHGVPVVAASGNAAV-DS-----------------CGITPANLPE--VITVAASN-------LE------------G-
Cre13.g585800   ---EGGYS----AAVNAMVDSLT-A-LGAVVVASAGNSNR-DT-----------------CAVSPASAQS--AV-----------------------------
Cre16.g675350   G---EPTTTPNAGRFIELATELVRK-HGVIFVASAGNAGP-AL-----------------TTVGAPGGTSSALFGVGAYV-----------------------
cons                          .      :.    *  .*.: **                           .        .

Cre02.g076950   QGRDSSPSSAVTASSPPPSPPPPSPAPPGSPKPGNSGNTPAKSPSPPPFPPPPSPSPPTGKPPPSPPPPSPPPPAPPPPSPPPPSPPPPSPPPPAPPIKKW-
Cre07.g329500   ------------------------------------------------------------------------------------------------------
Cre10.g459450   -----------------------------------KFNTSAR--RPPPLQPPL---------------------A-------SPRPLPPLQPPPREPMYS--
Cre13.g585800   ------------------------------------------------------------------------------------------------------
Cre16.g675350   --SP---------------------------QLAAAGH----------------------------------------------------------------
cons

                                                                                                              &
Cre02.g076950   (47 aa insert)-SSGSSGNSGSI-(84 aa insert)--LASPFSSWGPTPDLHIEPMLAAPGGAVLSTVPIVTRADGSRSTGYAYLSGTSQSAPYVAA
Cre07.g329500   -----------------GSSNRDD-------------------SLSSFSNVGGCVF------TFGPGGSIVSAYNTADNA-------EATMSGTSMATPHVSG
Cre10.g459450   -----------------------------------------------WSNTGKCVD------LFAPGVEIFGACGGKDRCPAVTASAYTWASGTSMAVPAVAG
Cre13.g585800   ---------------AVAAVEADG-------------------TRWYRSNWGSCVD------LFAPGVSLLAAVPWTDSA-------SGLKTGTSMAAPFASG
Cre16.g675350   ---------------SVLEAPAEG-------------------LQYNWSSRGPTSDGHTGVAFSAPGGAIAPVPQWTQQR-------RQLMNGTSMSSPNACG
cons                                                            *. *            .**  :  .                  .*** : * ...
```

```
Cre02.g076950   AAALYLQANKA---RQVSAAQVEAALVLTAKPLPDLRPTAAAVDAASAASAPSPATAAAGTAAATPASVLRVG--A-G
Cre07.g329500   VAAMILQAYPGASPATVRDKLAQAAVG-ITFSS------------------------------SSP---SRFL--NA-
Cre10.g459450   AAALYLESHPDASPREVAAALTRGATL-GALQD------------------------------------PRMRPGTPN
Cre13.g585800   VAALYLESHPGATPGDVAQRLLRSALA-GAVAD------------------------------------DRYG--Y-D
Cre16.g675350   GIALLLSGLLA-TGGALAPHRLRRALENTATPL----------------------------GGGAPDAVLTYG--R-G
cons              *: *..        :     . *    :
================================================================================================
```

MSA *The multiple sequence alignment result as produced by T-coffee.*

T-COFFEE, Version_11.00.d625267 (2016-01-11 15:25:41 - Revision d625267 - Build 507) Cedric Notredame SCORE=79

*BAD AVG GOOD * (transitive consistency score) color code goes from 0 (blue) to 9 (red).

Cluster 3: Shared region between oxyanion residue and S of catalytic triad varies from 246 to 570 aa in length; Cre03.g170300 (Pro-rich 45 amino acid insert between H and oxyanion residue); Cre03.g190250; Cre05.g242100; Cre05.g242750; Cre05.g242856; Cre09.g406700; Cre19.g750447.

```
                         &                                                                                   &
Cre03.g170300   GRGQVLGISDTGVDMD-SCYFFDPEIPDVWRRLVPDSK-----------------FKGAKVFNSDTHRKIKSYYAY--TDFGD--------ADGHGTHVSGIA
Cre03.g190250   GEGEVVGVGDTGLDLD-HCFYADPRFSNY----AALLK------------------GAPRRFRTPAHRKVVQYVVFKGAKFGDD-------GDGHGTHVCGSV
Cre05.g242100   GTGEVVGVTDTGVDMT-SCYFYDSQYGPALAALAANST------------------PGPFTLPMPAARKVVAYSVVQGAAYGDP------GSGAHGTHTSGTV
Cre05.g242750   GTGEILGSGDTGVDLD-NCYLSDPRYDSGA--VAGQLVNVTETWTSSNGNGTVWWRPDDRVWRPSDHRKLVQYYLPAGCVYGDNPA---QGTAGHGTHTAGSM
Cre05.g242856   GSGEILGSGDTGVDLD-NCYLSDPRYDSGA--VAGQLAAVTTQFLFSQGG-GYYARPGDRVWRPSDHRKLVQYYLPADCVYGDNPA---QGTAGHGTHTAGSM
Cre09.g406700   GQGQIVGVSDTGADVSGHCLLRDRRFPGRS--LSGQLS------------------GMPLRYQAPGHRKIPYYYLPAAPGESTDPSYFLDYAFGHGSHVVASL
Cre19.g750447   GRNQTVGCGDSGLDVA-NCYFYDPAVPFM-QNVKTD-------------------SNGMQYFLSSTHRKLRYYLAV-VQDLSD--------SYGHGTHVSGTL
cons            * .: :*  *:* *:   *   *                                            **:  *        .           .**:*. .

Cre03.g170300   LGACYLSPANASVPLETRSTNSSPPSPRPPSPRPPSPRPSPPPLKLKTKKAKKPPPPIPRPATPGTDGTGNGGGNVTNRSAAAGELLTNPANYVIDDAVGIAP
Cre03.g190250   AGAAMP--------VD---------------------------------------------------------------------G-NGTSDFVLQFGTGGAP
Cre05.g242100   AGAVPA--------GG-----------------------------------------------P-------------------GGPSGLGSGYRLDNATGAVP
Cre05.g242750   VGALLA--------LQ---------------------------------------------LAPGYNG--------SVLSGAAGEA-AAYGPTVLDAGSGAAP
Cre05.g242856   VGALLA--------LQ---------------------------------------------LAPGYNG--------SVLGGAAGDA-AAYGPTGLDAGSGAAP
Cre09.g406700   VGSVELP-------VV-------------------------------------------------------------------GTAPGVPGPFRDNNYTGAAP
Cre19.g750447   VGSRFDRA------------------------------------------------------------------------------S-ALSPEPLDAATGQAP
cons             *:                                                                                       .    :   * .*
```

(oxyanion residue) $

```
Cre03.g170300   GARLAFFDIMNKKK-------EYLL---PS----YDDLLTHQLESGVLVQSDSWGNKQT--FWYNGLCHQLDEVTWRHPELLVLTAAGNDGNMQPPPANGTVS
Cre03.g190250   RARLTFNDIGGTG-------GNLIN---IDN--VDELYLPFHKNVGAAITSDSWGPMWS---NYDGLARRFDSFLWRNPDMTSFIAAGNSGDDNTR-VGGTVD
Cre05.g242100   GARLSVIDISTAG-------GGLTV---PSP--MDQTLLQVHASAGVSITSDSWGVLSR---DYTAAANDFDRYLWSHGDHVALVAAGNYGQSPGL-RSGQVT
Cre05.g242750   RAKLSVVDISLPLV-RGDPEAGLQT---PEP--VDDVYLRLHYEVGARVTSDSWGTDIN---IYEETAQAYDAFLWRNPDSIAFIAAGNNGSDALT-PGGSIG
Cre05.g242856   RAKLSFVDISLTAAAAGGLQGALGP---PEP--VDDVYLRLHYDAGARITSDSWGAVAN---IYDDFAQAYDAFLWRNPDSIAFIAAGNNGSDALT-PGGSIG
Cre09.g406700   MARLSFADLATSS-------ASLTGLSYPGDYEFEEQTFGRHYGNGARLFSFSWGVSDSSGLSYGSWGAAMDDYLWRHPETLVVAAVGNNGANGY---MSTVQ
Cre19.g750447   AARIAFMDLSRDKG------DGVWT---PGD--LGRSYFNLTYRVGARVHSDSWGSDLT---AYDSMAAALDRFTWNNPDFLSIFAAGNYGTAS-GA-TTTVT
cons             *:::. *:                             :      *. : * ***        *       *   * : :   . *.** *          :


Cre03.g170300   NTLSVGSTLSLTASSTNGGALTLHVWTGGPEPPPEDPALVAASLPAEDAAALMSGDT------------AALS------------------APANAKALYG--
Cre03.g190250   NVVGVGVGYKYPAGVSFA-T-LLKIRGYQPS---------------SAG------D--VR-------------------------------SPANAK------
Cre05.g242100   NVIAVGSCLHSPPFTFYY-P-FYTVRGRRLG---------------SGQ------AWSQQVWPIECRDVGARDFLTGVGGPSGRNATLELVHPAAAKAVSGWS
Cre05.g242750   NIVAVGATYRVPAELGIGPQ-VLEVRGSTPG---------------TDG------STPTE-------------------------------TPATAK------
Cre05.g242856   NPVAVGATWRVPAELGLGAR-LLQVRGYNPA---------------ADLD----ASTSTE-------------------------------APATAK------
Cre09.g406700   NVLSAGASVNHPRDENPWANQWLPLFTYRDG---------------QGQ-----T-QQQA-------------------------------TPGSAK------
Cre19.g750447   NCIAAGATPSMGTGDRP-GAYGVTVYNL---------------------------------------------------------------SPAVAK--TA--
cons            * :..*                  :                                                            *. **


Cre03.g170300   SSVISALAA-QAAAAAQAAASGGGSSGDSSIGSSSSDGSAGFDGAVAALVDISFGGRKAPAAATSSPPPAGAAV-KAADDE-(50 aa insert)-EGLTLE
Cre03.g190250   ----------LFDLALQPA----------------------------------ETGG----------LPYLSSV-VSATSE--------------------TL
Cre05.g242100   LQPRPPPDPATGTLAQQQG----------------------------------EDGG--------GELPA------PGPSP--------------------AP
Cre05.g242750   ----------VLRLSVYPM----------------------------------ENPD----------LPQLLNI-IPLGSQ--------------------VP
Cre05.g242856   ----------LFRLALYPE----------------------------------ENPD----------LPQLLRV-IPADSE--------------------VP
Cre09.g406700   ---------------VWVT---------------------------------ARGSS----------SATWRNL-VEDQPP--------------------FA
Cre19.g750447   T-----------------VNIGGGQGGARSV----------------RVVGAAFGGD----------LAAAVSAAAA--------------------NGGVVA
cons                                                                                 .


Cre03.g170300   AVAASPLNACA---RLNDSSSASNNNKTAADAASAGAAAPLPKLLIT------TRDG-----C---MPYVKAAAAAAAGAAALAIANDAPGPA----VA-IL-
Cre03.g190250   VGVSTPIDACE---PLYPEA----------LAHVP------GRVVLVS-----YSDR-----C---DVDTRARNLQDAGAAAMMMIHHSPNMEYT-ESPPLSN
Cre05.g242100   AGPAAFSSSCSLPPGL-VAS-----------GALA------GAVAVAP-----ASPN-----C---SLVEQVLALQRAGAAAVLLGYTEPSNP---PTPVLVS
Cre05.g242750   LVVADPPDACT---PLTNPA-----------AEVR------GAVVLA------VRGE-----C---WFYQKVLHVRAAGGGAVIFVNNVPEMMDSPPAPVYPN
Cre05.g242856   LVVADPPDACT---PLTNPA-----------AEVR------GAVVLA------VRGE-----C---WFYQKVLNVRAAGGGAIIFVNNVPEMMDSPPYPIYPN
Cre09.g406700   VVLASPLHACT---PLQPVPGSAS------PSQYQ------GAIVIIDPVADTSPDGTLPPDCQGVLDSDRASYAMEAGAVAVWFVWRYYRTVL---SEAYVI
Cre19.g750447   LAAATPADACI---GLSNAGGV------------R------GRVLLV------QRGR-----C---YFSDKARAAAAAGATAVIVYNDKQDGYFLMEAPDAS-
cons               :    :*     *                            : :                *       :.     **. *: .
```

```
Cre03.g170300   -----VNPTGSTLPAAALTQDLGARIFAALAAG--QR-V-FARA-SWQ---PQGPYDHVPAFSGWGPTNDGRVKPDIVAPGWRVVSAYSD----FVSAGVS--
Cre03.g190250   -----SDGPAIRIPVSSIPQVMGGWLVGNVTAG--VE-L--RLT-WQD---YPQSVGDVVRWSGYGPTRDGRIKPDVIAPGYDIVSAKAVD-------GIT--
Cre05.g242100   SPSAALNSTGVTIPVGYITQSLCSWLLGNMSAS--SRPV--TFSVSLS---ASQGINSVASFSSAGPALDGRVKPDLVAPGFQLTSAWKTGAGAGAGAGTT-Q
Cre05.g242750   -----SNYYAAKMATSVISQGLGQWLLGNASAG--VE-L--SLA-NTV---KNAGSDTVAFFSSYGPMADGRIKPDIVTPGVLITSAGAKG-------GIT--
Cre05.g242856   -----SNSYAANMATSVISQGLGQWLLGNASAG--VL-M--TLA-NIV---EAAGPDTVAYFSSYGPMADGRIKPDVVAPGLYITSAGARG-------GIT--
Cre09.g406700   -----QGVSVIPLPYSIITREQGDWLQQVMTDPSHSE-F--RFQ-NITYPLVNGGEESLAFFSSYGPTPDGRFKPDLVAPGQRLISASSGA-------GLRAV
Cre19.g750447   -----GGSGSGLVPMGSVPASTGRWLLAAAAAG--NTAVSIRRA-ADV---APLAFDDVAAYSSFGPTTDGRIKPDITAPGHLLS-ANANPT--LAPDGAV--
cons                  .     :. . :.      :    :       .                   :  :*. **  ***.***: :**  :  *           *

                             &
Cre03.g170300   D-GC-AV-RMTMEGTSMSAPLVAGAALLARQYFESGHYPAGRTDAPEAAPFSPSGVLLKAVLIGGAYDMSRSGGLSFSTLQPLLPAPSAYQGFG
Cre03.g190250   GGQC-NDETQEMSGTSMATPLAAGSAALLRQYLRSGFYPGGWPDTQDSAPFKPSGILVKALVIAGAKS--LQGGWALQVARLLSTAPDYYQGWG
Cre05.g242100   GDTC-GVSVAARSGTSMSTPTAAGHTALVRQYLRTGFYPTGSPADAAAAPFTPSGMLLKAMVIAGAKS--MEGGEAMATRAPLGPPPDAYQGWG
Cre05.g242750   GGAC-SPAQANLSGTSMATPHAAGHTALVRQYLRTGFYPTGSPADAAAAPFTPSGMLLKAALIAGAKS--LQGGLAMALGIPMGPPPDAYQGWG
Cre05.g242856   GGAC-SPAQANLSGTSMATPHAAGHTALVRQYLRTGFYPTGSPADAAAAPFTPSGMLLKAAIIAGAKS--LMGGLAMALGIPMGPPPDAYQGWG
Cre09.g406700   ETSC-SLEMRSLAGTSMSTPVVAGNLAIARQYLTEGWYPAGWRGAPDSAPFVPSGPLLKAIAIAGAKS--LQGGLARAPGQLMGTPPDGYQGWG
Cre19.g750447   P-SCGAGATRILSGTSMATPVIAGSALL------------------------------------------------------------------
cons               *         ****::*  **   :
```

Supplemental Fig.2. Alignment of S8 domains of 21 *C. reinhardtii* family members. Phytozome identifies 21 proteins that contain the subtilisin-like S8 domain. A phylogenetic tree constructed using all 21 S8 domains, identified subclusters, which are marked. For each subcluster, catalytic core sequences were aligned using T-Coffee. Catalytic triad residues are marked, along with the oxyanion residue.

Supplemental Fig.3. Alignment of two M14 CPB-like *C. reinhardtii* and human proteins. Alignment of *C. reinhardtii* CPB-like enzymes CPZ2(Cre06.g309450) and CPZ3 (Cre07.g335900) with the three M14 domains of human CPD (CPD1, CPD2, CPD3) and the M14 domain of human CPE. M14 catalytic core sequences were aligned using T-COFFEE, Version_11.00. 8cbe486 (2014-08-12 22:05:29 - Revision 8cbe486 - Build 477). Cedric Notredame. SCORE=79. * BAD AVG GOOD* TCS (transitive consistency score) color code goes from 0 (blue, bad) to 9 (red, good).

```
CPD1     AHIKKAE---------------------------ATTTTTSAGAEAA--EGQF-----------------------DRYYHEEE-LES--
CPD2     ------Q---------------------------PI------Q--PK--D--F------------------------HHHHFPD-MEI--
CPD3     ----TTK---------------------------EF--------ETLIKD-LSAENGLESLMLRSSSNLALALYR---YHSYKD-LSE--
CPE      ------L---------------------------QQ-----ED--GI--S--F------------------------EYHRYPE-LRE--
CPZ3     -WTSAAQGLRRPDADLPATSELDNGGTSAQQLGSAL--------RPL--SDTF---------------------VATRNGQLTDGQEFAS
CPZ2     -YPTFA-------------------------------------------------------------------------------Q-LSE--
cons                                                                                         :

CPD1     -----AL----REAAAAGLPGLA--------------RLFSI-GRSVEGRPLWVLRLTAGLGSLIPEGDAGPDAAGPDAAG-PLLP-GRP
CPD2     -----FL----RRFANE-YPNIT--------------RLYSL-GKSVESRELYVMEISD------NPGVH--E------PG-------EP
CPD3     -----FL----RGLVMN-YPHIT--------------NLTNL-GQSTEYRHIWSLEISN------KPNVS--E------PE-------EP
CPE      -----AL----VSVWLQ-CTAIS--------------RIYTV-GRSFEGRELLVIELSD------NPGVH--E------PG-------EP
CPZ3     PPPGEFVGDEWARYHN--HDEMVRLLQDVVNNSNGACTLDELDPPSVEGRVLYAVVASPR-----NP-----------AGG--AVLPRP
CPZ2     -----YL----A---------------LRVAGSGLRCSLASA-GKSVQGRDLWAVTIGD------PAGVYYPD------PTNPDVPFPKA
cons          :                        :       *  :  *  :   :                   .          ..

CPD1     QVKLVGNMHGDETVSRQVLIYLARELAAGYR---------RGDPRLVR------LLN-TTDVYLLPSLNPDGFERAREGDCGFGDGGPSGA
CPD2     EFKYIGNMHGNEVVGRELLLNLIEYLCKNF---------GTDPEVTD------LVH-NTRIHLMPSMNPDGYEKSQEGDSI-------SV
CPD3     KIRFVAGIHGNAPVGTELLLALAEFLCLNY---------KKNPAVTQ------LVD-RTRIVIVPSLNPDGRERAQEKDCT-------SK
CPE      EFKYIGNMHGNEAVGRELLIFLAQYLCNEYQ--------KGNETIVN------LIH-STRIHIMPSLNPDGFEKAA-SQPG-------EL
CPZ3     SFTWIGNMHGDETANRELLLRLAAGLCN---------G-----ELADSDARWKALQGSTTVRIIPTMNPDGYERR---------------
CPZ2     RAAYIGVMHGDEKGHISAVLRLVGELCDPLSEPKFAPGGVLDSNVTD------LL-GSTVLYVLPLMNPDGYTAT---------------
cons       :.  :*:       .  ::  *    *.              :.       :   *  :  ::*  :****

CPD1     ----SGRDNSRGRDLNRSFP-----------DQF-------------------STGEPPA--LD--EVPEVRALIE-WIRRNKFVLSGNLH
CPD2     ----IGRNNSNNFDLNRNFP-----------DQF------V-----------QITDPTQ--------PETIAVMS-WMKSYPFVLSANLH
CPD3     ----IGQTNARGKDLDTDFT-----------N------------------N------AS--------QPETKAIIENLIQKQDFSLSVALD
CPE      KDWFVGRSNAQGIDLNRNFP-----------DLDRIVYVNEKEGGPNNHLLKNMKKIVDQNTK--LAPETKAVIH-WIMDIPFVLSANLH
CPZ3     -----TRWNANKVDLNRNFWSVEYPYAKPTLDQA------RKRDRYGATMYPAADMWSKVG-NFTLQPEAVAVSR-YLAAAPPDLSANLH
CPZ2     -----QRYNANGVDLNRNFYTSAFPFAMPTAADG-----YALQPGTSNALYNAAADWTDNGGGGAHEPETQAVMS-WLASVRPHVSADLH
cons          : *:.   **: .*                                           **. *:    :       :*   *.

CPD1     GGSVVASYPFDDSPEHKATGIYSKTSDDEVFKYLAKAYASNHPIMKT-GEP-H-CP-GDEDETFKDGITNGAHWYDVEGGMQDYNYVWAN
CPD2     GGSLVVNYPFDDDE--QGLATYSKSPDDAVFQQIALSYSKENSQMFQ-GRP---CKNMYPNEYFPHGITNGASWYNVPGGMQDWNYLQTN
CPD3     GGSMLVTYPYDKPV--Q--TVENKE----TLKHLASLYANNHPSMHM-GQP-S-CP-NKSDENIPGGVMRGAEWHSHLGSMKDYSVTYGH
CPE      GGDLVANYPYDETRS-GSAHEYSSSPDDAIFQSLARAYSSFNPAMSD-P-NRPPCRKNDDDSSFVDGTTNGGAWYSVPGGMQDFNYLSSN
CPZ3     GGALVANYPLDACD-SVGALTNCPTAEEPLPRQLAAAYAVHNPNMSAQGT-----------APFRAGTVQGAAWYPVLGSMQDWVYHHLD
CPZ2     GGALVGSYALDACD-SRGALLDCPSPEAPLPGYLANVYSMNHPSMRF-SWG-E-VQ-ASRQVQFFNGTTQGATWYPAIGTIADWLHHTYR
cons     ** :: .*. *                      :*   *:  :. *                  :  *  .*. *:   * : *:

CPD1     CFEITLELSCCKYPPA-SQLRQEWENNRESLITLIE-KVHIGVKGFVKDSITGSGLENATISV-AG--INHNITTGR--FGD-FYRLLVP
CPD2     CFEVTIELGCVKYPLE-KELPNFWEQNRRSLIQFMK-QVHQGVRGFVLDATDGRGILNATISV-AE--INHPVTTYK--TGD-YWRLLVP
CPD3     CPEITVYTSCCYFPSA-ARLPSLWADNKRSLLSMLV-EVHKGVHGFVKDKT-GKPISKAVI-VLNE--GI-KVQT-K--EGGYFHVLLAP
CPE      CFEITVELSCEKFPPE-ETLKTYWEDNKNSLISYLE-QIHRGVKGFVRDLQ-GNPIANATISV-EG--IDHDVTSAK--DGD-YWRLLIP
CPZ3     RLMLTLELHEVKDPPA-TALDDLWGQNSAAFLRIME-LAGMGLRGRVLDQQT-RAPLAATITV-TSPASSRPADA-DAARGGYFYKPMAP
CPZ2     RHMLTLELHYYKYAMFLNDKLNMYATNRPSMLRLAGVGAHMGLRALLRDAAS-GAALAATVTP-QQPAGAWAPEV-E--SGGLVWKMAMP
cons         :*:      .         :  *   :::      *::. : *        *.:                  *.          *
```

```
CPD1    GT-YNLTVVL-------TGYMPLTV-TNV--------------VV-----KEG----PATE-VDFSL------------------R--PT
CPD2    GT-YKITASA-------RGYNPVTK-N-V--------------TVK---SEGA------IQ-VNFTL--V----RS---------STDSN
CPD3    GV-HNIIAIA-------DGYQQQHS-Q-VFVHHDAASSVVIVFDTDNRI---------------FGL--P--------------------
CPE     GN-YKLTASA-------PGYLAITK-K-V--------------AV----PYSP------AAGVDFEL--ESFSERKEEEKEELMEW--WK
CPZ3    GVSYDITVQPYRPNGGAIKYLPIQLKG-L----------RLGVAADRVTAEGSAVTGAALR-IERTLLAT----RA--------------
CPZ2    GVTYSGTIYPYDPADSSIAYEPIPY-S-F----------VVPYTWDDVL---SKVPRANLS-IARVFRAV----RV--------------
cons    *  :.              *        .                                     :

CPD1    VTSVIPDTTEAVSTASTVAIPNIL----------------SG----TSSSY
CPD2    NESKKGK----GASSSTNDASDP----------------------------
CPD3    -----------------------------------------RE--------
CPE     MMSETLN----F---------------------------------------
CPZ3    --------------------------PA--------ANKKKKMLRATM---
CPZ2    ------G----GSQASTATAPPPQQPPPPNAPQPPLQQQQQRRRRR-----
cons
```

Supplemental Fig.3. Alignment of two M14 CPB-like *C. reinhardtii* proteins with human proteins homologs. The *C. reinhardtii* proteins most homologous to human CPD and CPE were identified using BLASTp. Catalytic core sequences were aligned using T-Coffee. Zinc-binding residues and the active site are marked.

- You list O, C, M, Sp in supplemental Table 3 under Pred Algo

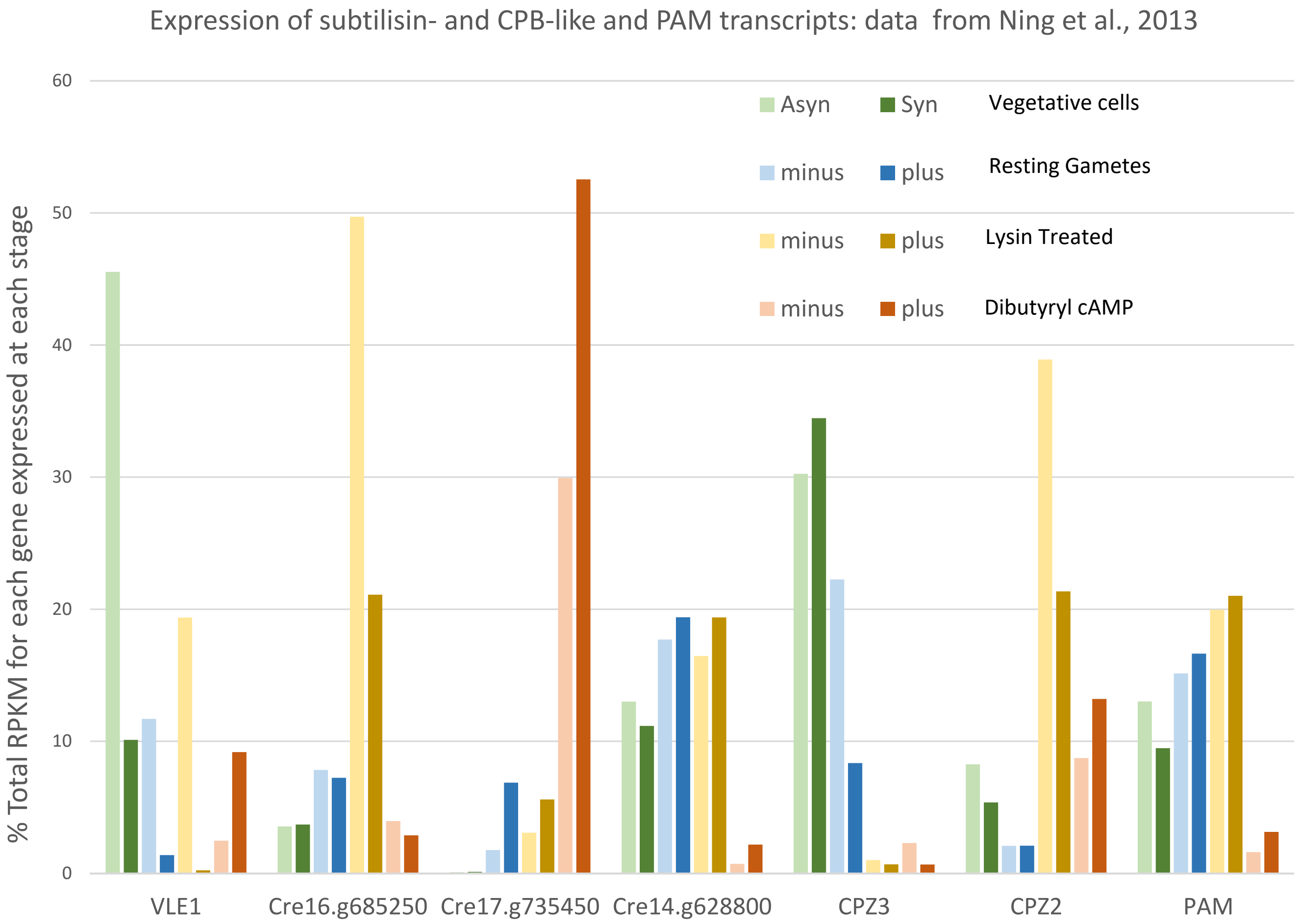

Supplemental Fig.4. Expression of *C. reinhardtii* subtilisin-like, CPB-like and PAM transcripts. Relative transcript levels (% total RPKM for each transcript in *plus* and *minus* mating type cells at each stage examined) are shown for the *C. reinhardtii* genes encoding proteins most like human furin, PC1, PC2, CPD, CPE and PAM [19]. Stages examined in this study were: Vegetative cells (Asy, asynchronous, and Syn, synchronous); Resting Gametes; Lysin-treated gametes; Dibutyryl-cAMP treated gametes.
